# Optimized Epigallocatechin Gallate Delivery and Adipogenesis Inhibition through Fluorescent Mesoporous Nanocarriers

**DOI:** 10.34133/bmr.0053

**Published:** 2024-07-16

**Authors:** Taelin Kim, A. Yeon Cho, Sang-Wha Lee, Hyun Jong Lee

**Affiliations:** School of Chemical, Biological and Battery Engineering, Gachon University, 1342 Seongnam-daero, Seongnam-si, Gyeonggi-do13120, Republic of Korea.

## Abstract

Epigallocatechin gallate (EGCG), a naturally occurring compound known for its multiple health benefits including antioxidant, anti-inflammatory, cancer preventive, and weight management effects, faces challenges due to its inherent instability and limited bioavailability. To address these limitations, our study pioneers an investigation into the unique behavior of EGCG, revealing its degradation into epicatechin (EGC) and gallic acid (GA) during the drug delivery process. In this research, we use fluorescent mesoporous silica nanoparticles (FMSNs) as a sophisticated delivery system for EGCG. This innovative approach aims to not only enhance the stability of EGCG but also regulate its sustained release dynamics to enable prolonged cellular activity. To comprehensively evaluate our novel delivery strategy, we performed assays to assess both the antioxidant potential and its impact on lipid inhibition using Oil Red O. The results not only underscore the potential of FMSN-based nanocarriers for efficient EGCG delivery but also reveal groundbreaking insights into its enzymatic degradation, a previously unexplored facet. This research substantially advances our understanding of EGCG’s behavior during delivery and offers a promising avenue for improving its therapeutic efficacy and expanding its applications in health management.

## Introduction

Obesity, characterized by the excessive accumulation of body fat, is associated with several diseases, including metabolic syndrome, diabetes mellitus, cardiovascular problems, fatty liver disease, and cancer. This condition is characterized by chronic low-grade inflammation and a sustained increase in oxidative stress, which can lead to cellular damage and a deficiency of antioxidant defenses. These factors contribute to the development of obesity-related complications [1]. Adipogenesis, the process through which adipocyte progenitor cells differentiate into mature adipocytes, plays a central role in determining the number of adipocytes in the body [2]. Therefore, understanding the molecular mechanisms involved in adipogenesis is critical for identifying potential therapeutic targets in the fight against obesity [3].

To harness the potential health benefits of natural compounds, epigallocatechin gallate (EGCG) has emerged as a prominent subject of scientific investigation [4]. EGCG, a catechin abundant in green tea, has shown promising properties ranging from antioxidant and anti-inflammatory effects to potential roles in cardiovascular health, cancer prevention, and weight management [5,6]. However, maximizing the efficacy of EGCG and overcoming its limitations require a deeper understanding of its optimal delivery to the human body. The limited bioavailability and easy hydrolytic degradation of EGCG pose significant hurdles because a substantial portion may not be efficiently absorbed by the body [7]. This limitation hampers the realization of its full therapeutic potential and necessitates the development of an efficient delivery system that can enhance its absorption, stability, and targeted delivery to specific tissues or cells.

Flavonoid catechins have beneficial biological properties, such as antioxidant, anti-inflammatory, and neuroprotective effects [8]. However, Dube et al. [8] reported that a stabilization strategy is required for the long-term use of chemically unstable catechins. Xu et al. [9] found that temperature and pH significantly affect the epimerization and degradation of EGCG in an aqueous system. Dai et al. [10] reported that nanocarriers improve intestinal stability and prolong the residence time of EGCG. However, inappropriate nanoparticles (NPs) formed by molecular interactions can induce EGCG deformation owing to excess ions and changes in gastric pH [10]. Researchers are actively investigating various strategies, including encapsulation, chemical modification, and combination with other compounds, to protect and stabilize EGCG and improve its safety and efficacy as a potential therapeutic agent. Therefore, it is important to carefully select a nanocarrier based on its intended application and thoroughly investigate the anomalous release kinetics associated with EGCG degradation throughout the release process.

The primary objective of this study was to investigate the release kinetics of EGCG-loaded fluorescent mesoporous silica nanoparticles (FMSNs) with antioxidant and adipogenic inhibitory activity. Specifically, we investigated the loading of EGCG into FMSNs and evaluated the anomalous release kinetics caused by its simultaneous degradation during the release process in our quest to improve the efficacy of MSNs. Polydopamine (PDA), a known enhancer of biocompatibility and cellular uptake, was incorporated into FMSNs. We measured the release patterns of EGCG loaded onto FMSNs and FMSNs@PDA and evaluated the radical scavenging activity. The release dynamics of 3T3-L1 cells were substantiated by examining cell viability and their ability to inhibit adipogenesis. The results of this study demonstrate an efficient EGCG delivery method with the potential to effectively manage obesity.

## Materials and Methods

### Materials

Ammonium fluoride (NH_4_F), cetyltrimethylammonium bromide (CTAB), tetraethyl orthosilicate, fluorescein isothiocyanate (FITC), 3-aminopropyl-trimethoxysilane (APTMS), dopamine hydrochloride (DA), Trizma hydrochloride buffer solution (Tris-HCl), (−)-EGCG, epigallocatechin (EGC), insulin, 3-isobutyl-1-methylxanthine, dexamethasone, and isopropanol were purchased from Sigma-Aldrich (St. Louis, MO, USA). Ethanol and gallic acid (GA) were purchased from Daejung Chemicals (Gyeonggi-do, Korea). Dimethyl sulfoxide (DMSO) was purchased from Duchefa Biochemie (Haarlem, The Netherlands). Hydrochloric acid was purchased from Samchun Chemicals (Seoul, Korea). Phosphate-buffered saline (PBS) was purchased from Bioneer Co. (Daejeon, Korea). Dulbecco’s modified Eagle’s medium (DMEM) was purchased from Hyclone (Washington, DC, USA), and bovine calf serum (BCS) was purchased from Thermo Fisher Scientific (Waltham, MA, USA). Penicillin and fetal bovine serum (FBS) were purchased from Welgene Inc. (Daegu, Korea). 3-(4,5-Dimethylthiazol-2-yl)-2,5-diphenyltetrazolium bromide (MTT) was purchased from Invitrogen (Carlsbad, CA, USA). Oil Red O staining kit was purchased from Science Cell (San Diego, CA, USA). 2,2-Diphenyl-1-picrylhydrazyl (DPPH) antioxidant assay kit was purchased from Gerbu (Gaiberg, Germany). All chemicals and reagents were used as received without further purification or modification.

### Synthesis of FMSNs

The synthesis of FMSNs described in this study included the following steps: FITC (2.5 mg) and APTMS (0.12 ml) were dissolved in ethanol (3 ml). The APTMS-FITC complex (A-F complex) was formed by stirring the solution at room temperature for 6 h, followed by refrigeration at 3 °C for 18 h. For the preparation of MSNs, a mixture of CTAB (0.15 g) and NH_4_F (0.4 g) was prepared in 100 ml of deionized water at 80 °C. The solution was stirred at 1,500 rpm for 1 h, after which 2 ml of tetraethyl orthosilicate was added dropwise to make the solution milky. The A-F complex was then added to the milky solution, and the reaction mixture was stirred at 80 °C for 24 h in the dark. During the process, the A-F complex was incorporated into the growing silica structure. The resulting yellow solution was centrifuged at 8,500 rpm for 8 min, and the collected particles were washed several times with water and ethanol to remove any remaining impurities. To remove the surfactant CTAB from the mesopores of the silica particles, the washed product was dissolved in ethanol and stirred twice in hydrochloric acid-added ethanol at 80 °C. This step ensured the removal of CTAB. Upon completion of the CTAB removal process, the yellow product was collected and dried at 60 °C for 24 h in the dark. The final products obtained from this process were characterized by their incorporated FITC dye and fluorescence properties.

### PDA coating of MSNs

**Fig. 1.**
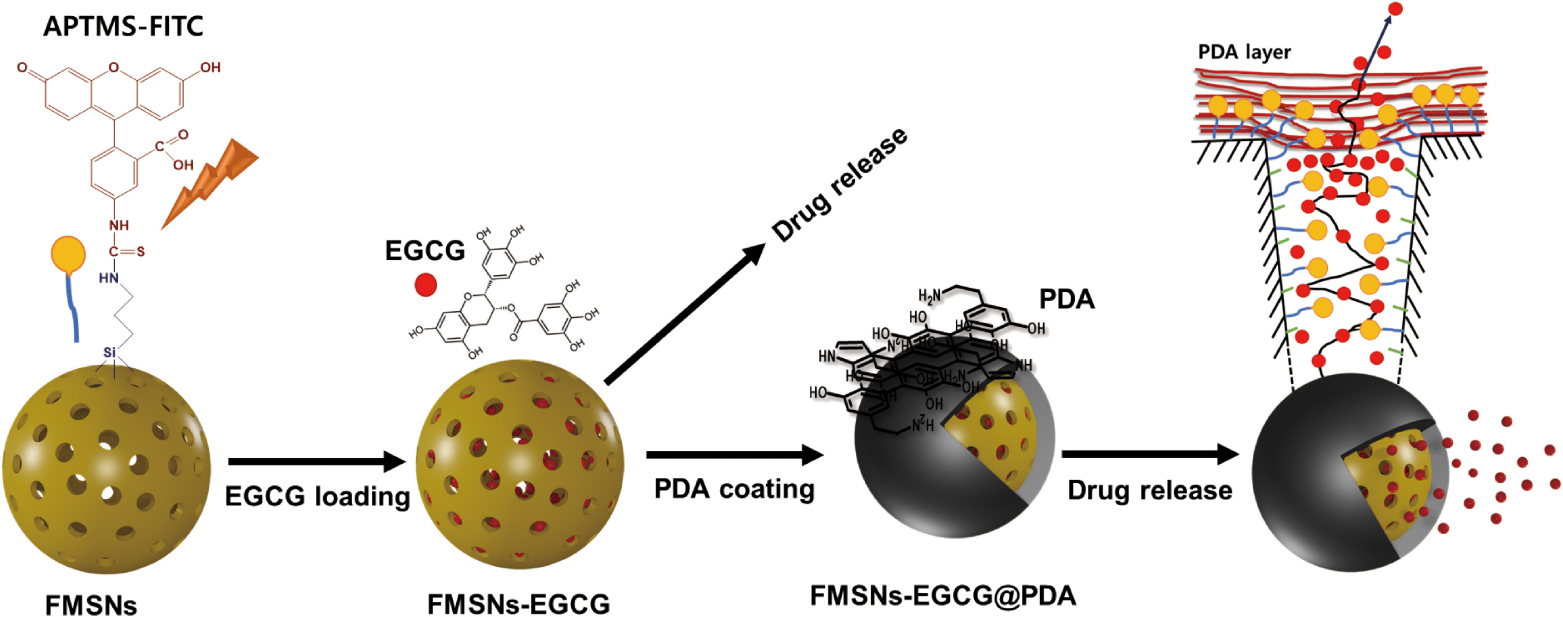
Stepwise procedures for preparing samples (FMSNs, FMSNs-EGCG, and FMSNs-EGCG@PDA).

A total of 100 mg of FMSNs-EGCG was dispersed in 10 ml of Tris-HCl. Ten milligrams of DA was added to this dispersion of FMSNs. DA can undergo oxidative polymerization under alkaline conditions to form a PDA coating [11]. The mixture containing FMSNs and DA was allowed to react at room temperature for 3 h. After the reaction, PDA-coated FMSNs loaded with EGCG (FMSNs-EGCG@PDA) were obtained by centrifugation, and the collected particles were washed with deionized water to remove any remaining impurities. The washed particles were then freeze-dried for 24 h. Finally, FMSNs-EGCG@PDA was stored in the dark to maintain stability. The synthesis procedure is shown in Fig. 1.

### Drug loading into FMSNs

Ns were dispersed in a DMSO solution containing the test drugs, including EGCG. DMSO, a common solvent, was used to dissolve various compounds. FMSNs and drug-containing DMSO solution were stirred for 24 h in the dark. After the incubation period, the drug-loaded FMSNs were obtained by centrifugation. To remove unincorporated drug molecules from the particle surfaces, FMSNs-EGCG was washed with deionized water. The washed FMSNs-EGCGs were lyophilized for 24 h. The amount of drug loaded into each particle is summarized in Table. The change in absorbance of EGCG in the solution before and after drug loading was evaluated, and the amount of drug loaded and the loss of NPs were calculated based on the standard curve. Figure S1A shows the absorbance of EGCG dissolved in DMSO (ranging from 0 to 100 μg/ml) by analyzing the absorbance at 274 nm. Figure S1B and C shows the absorbance of EGC (10 to 40 μg/ml) and GA (10 to 60 μg/ml), which are degradation products of EGCG, at 220 and 260 nm, respectively, with data analyzed by linear regression. The loading amount of EGCG in FMSNs-EGCG was 131 mg/g (mg drug/g particles). During the PDA coating process, FMSNs-EGCG showed a weight loss of 34.5%. The volume of all solutions used in the coating and washing processes was fixed at 10 ml to maintain experimental uniformity.

**Table.** Loading amounts of drug in FMSNs and FMSNs@PDA after surface modification

As-prepared sample	FMSNs-EGCG	FMSNs-EGCG@PDA
Initial amounts of drug in the solution before the drug loading process (mg/ml)^a^	25	25
Residual amounts of drug in the solution after the drug loading process (mg/ml)^b^	23.10	23.10
Drug loss amount in the washing solution after surface modification (mg/ml)^c^	0.59	1.05
Final loading amount of drug in the particle (mg/g)^d^	131	84.5 (loss 35.50%)

^a^ Amount of EGCG dissolved in DMSO before drug loading.

^b^ Amount of EGCG dissolved in DMSO after drug loading.

^c^ Amount of EGCG lost during the washing and coating processes.

^d^ Amount of EGCG loading per 1 g of particle.

### Drug release test

In the in vitro release test, the drug release from NPs was investigated under different pH conditions. The test was performed at pH 7.4, which is neutral (similar to blood and normal cells), pH 5.5, which is acidic (similar to endosomes), and pH 4.0, which is highly acidic (similar to lysosomes). In each case, 30 mg of NPs was suspended in PBS. The suspension was maintained at 37 °C, which is a typical physiological temperature. Stirring was performed throughout the assay to ensure adequate mixing and interaction. The absorbance of EGCG was measured at 274 nm using an ultraviolet-visible spectrophotometer (HP 8453, Agilent Technologies, Santa Clara, USA).

### Radical scavenging activity

The antioxidant capacity of DPPH was evaluated using a DPPH antioxidant assay kit according to the manufacturer’s instructions. Briefly, 100 μl of DPPH working solution in ethanol was thoroughly mixed with 100 μl of drug solution at specified intervals (0, 6, 24, 48, and 72 h). After incubation for 30 min at 25 °C, spectrophotometric measurements (Microplate Spectrophotometer, Epoch) were performed at 517 nm. DPPH radical scavenging efficacy was calculated using the following formula:

DPPH radical scavenging activity (%) = [(absorbance of control − absorbance of sample)/absorbance of control] × 100.

### Cell culture and viability assessment

3T3-L1 fibroblasts were obtained from the Korea Cell Line Bank and maintained in an incubator at 37 °C with 5% CO_2_. The cell culture medium was DMEM supplemented with 1% penicillin and 10% BCS. After seeding 8 × 10^3^ cells in a 24-well plate, the cells were allowed to grow to confluence, and experiments were initiated. The MTT assay was performed to evaluate cell proliferation as a function of drug concentration. After seeding 3T3-L1 cells, different drug concentrations (2.5, 5, or 10 μM) were applied, and the culture medium was refreshed every 2 days. On days 1, 4, and 7, MTT solution dissolved in DMSO was added, and the absorbance at 540 nm was measured using a spectrophotometer.

### Adipocyte differentiation induction and Oil Red O staining

3T3-L1 cells were induced to differentiate using DMEM supplemented with 10% FBS along with 10 μg/ml insulin, 0.5 mM 3-isobutyl-1-methylxanthine, and 1 μM dexamethasone, agents known to induce adipocyte differentiation. For the first 4 days, the cells were cultured in DMEM containing 10% FBS and 10 μg/ml insulin, with a media change on the second day. To ensure complete differentiation, the cells were maintained in DMEM with 10% FBS for an additional 2 days, starting on the fourth day of differentiation.

After 8 days of induced differentiation of 3T3-L1 cells into adipocytes, the medium was removed, and the cells were subjected to Oil Red O staining. After 2 washes with PBS, the cells were fixed in 4% paraformaldehyde solution. Cells were then exposed to 60% isopropanol solution, incubated for 5 min, and stained with Oil Red O working solution for 15 min. The Oil Red O working solution was prepared by diluting the Oil Red O stock solution with distilled water in a 6:4 ratio. After staining, the samples were rinsed thrice with distilled water and observed under a microscope. To quantify the amount of lipid staining, the cells were washed 3 times with 60% isopropanol, and lipids were extracted with 100% isopropanol. The absorbance was measured at 490 nm using a spectrophotometer.

### Statistical analysis

Data are presented as mean ± SD. Unless otherwise noted, all experiments were performed in triplicate to ensure reliability and reproducibility. For multiple group comparisons, statistical analysis was performed using one-way analysis of variance (ANOVA), and a *P* value of <0.05 was considered statistically significant. GraphPad Prism 9 software (San Diego, CA, USA) was used for statistical calculations.

## Results

### Physicochemical properties of MSN-based nanocarriers

**Fig. 2.**
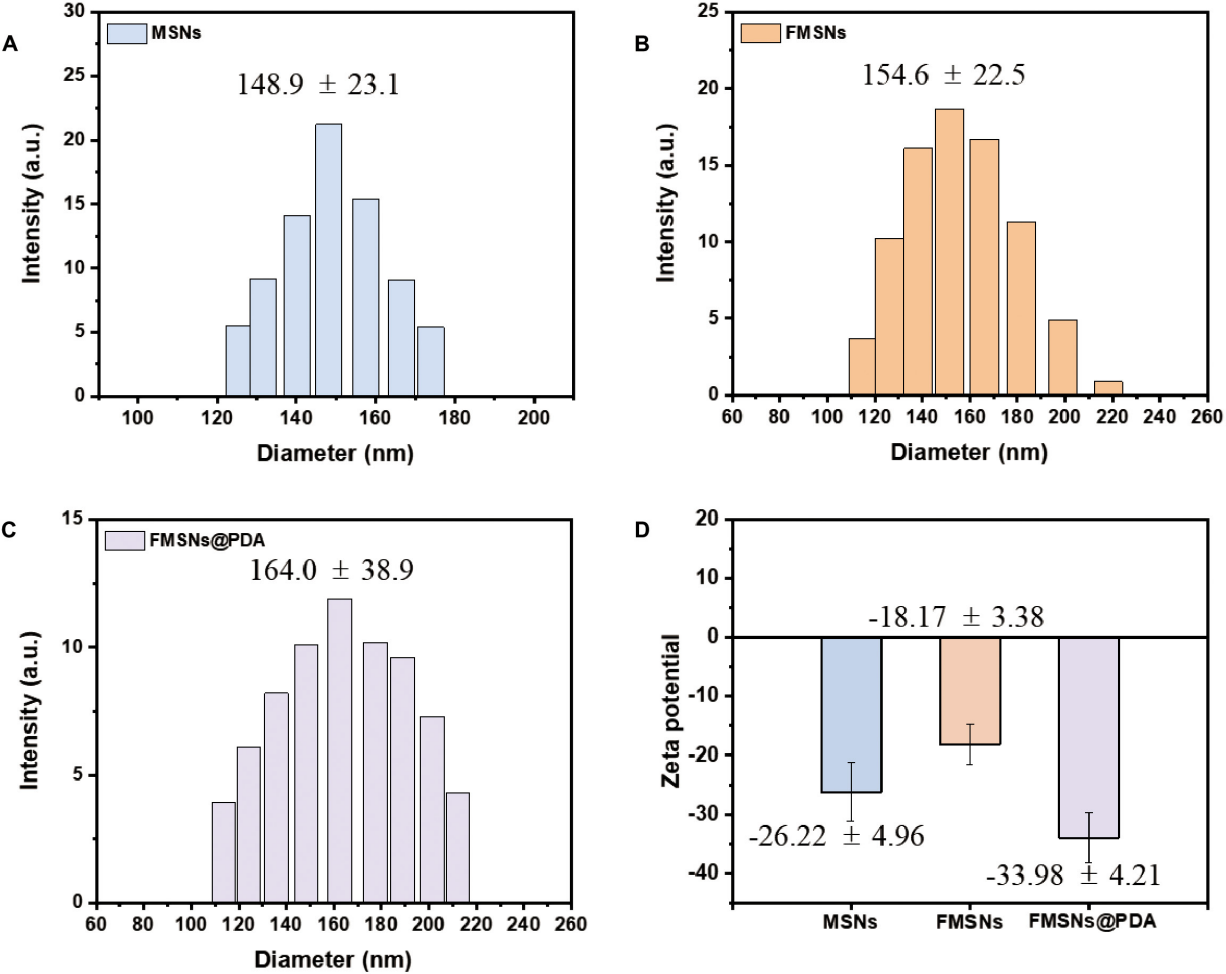
Particle size of (A) MSNs, (B) FMSNs, and (C) FMSNs@PDA and (D) zeta potential of samples in PBS (pH 7.4) using dynamic light scattering.

The surface modification and colloidal stability of the NPs were evaluated by measuring their hydrodynamic size and zeta potential using electrophoretic light scattering (ELS; ELSZ-2000, Otsuka, Tokyo, Japan), as shown in Fig. 2. The size distribution of the samples in PBS (pH 7.4) at a concentration of 0.5 mg/ml was measured. The hydrodynamic diameter of the NPs was measured (Fig. 2A to C): MSNs (148.9 ± 23.1 nm), FMSNs (154.6 ± 22.5 nm), and FMSNs@PDA (164.0 ± 38.9 nm). The size of the NPs gradually increased due to stepwise surface modification. The zeta potentials were −26.22 ± 4.96 mV for MSNs, −18.17 ± 3.38 mV for FMSNs, and −33.98 ± 4.21 mV for FMSNs@PDA, indicating successful surface modification and high colloidal stability (Fig. 2D).

**Fig. 3.**
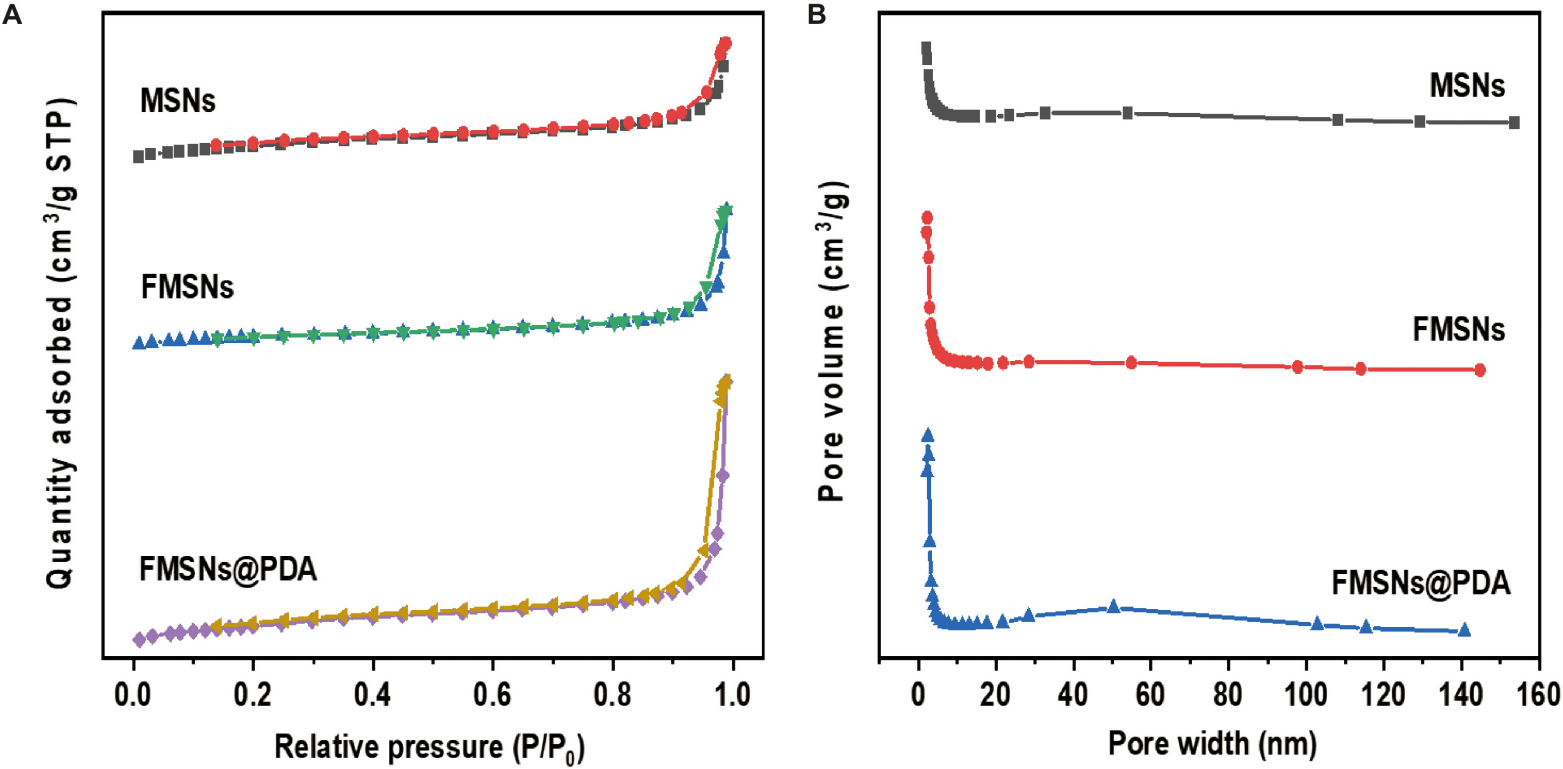
Nitrogen adsorption/desorption isotherms (A) and pore size distribution using BJH analysis (B) for as-prepared samples (MSNs, FMSNs, and FMSNs@PDA).

The Brunauer–Emmett–Teller (BET) adsorption isotherm using N_2_ adsorption and desorption on the samples (MSNs, FMSNs, and FMSNs@PDA) and their pore distribution according to Barrett–Joyner–Halenda (BJH) are shown in Fig. 3A and B, respectively. The MSNs exhibit a BET surface area of 84.5 m^2^/g and a pore size of 9.8 nm, which represent the specific surface area and average pore size for MSNs. FMSNs exhibit an increased BET surface area of 127.4 m^2^/g and a larger pore size of 17.1 nm compared to the MSNs. This increase is attributed to the conjugation of the A-F complex, resulting in additional surface coverage and pore enlargement. After PDA coating, the surface area further increases to 156.6 m^2^/g, accompanied by a slight reduction in pore size to 16.7 nm, indicating a pore-blocking effect of the PDA layer.

**Fig. 4.**
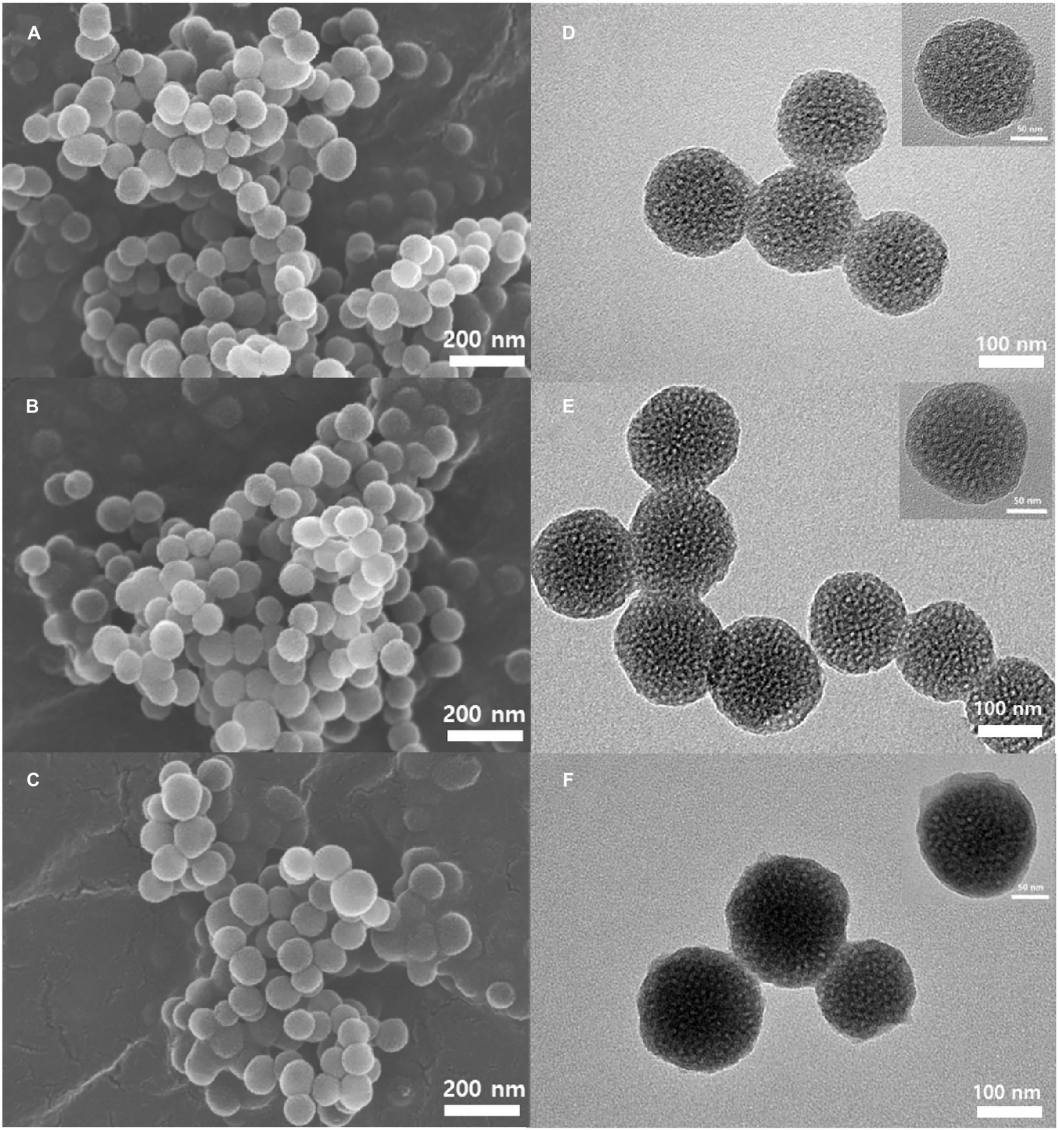
SEM and TEM images of (A and D) MSNs, (B and E) FMSNs, and (C and F) FMSNs@PDA.

The as-prepared samples were characterized by scanning electron microscopy (SEM; SU8600, Hitachi, Tokyo, Japan) and transmission electron microscopy (TEM; H 7600, Hitachi, Tokyo, Japan). Figure 4A to C shows the surface morphologies of the as-prepared samples (MSNs, FMSNs, and FMSNs@PDA) obtained using a SEM. All the samples showed spherical shapes with uniform size; however, there were no distinct differences in the surface morphologies of the samples irrespective of different surface modification steps. Figure 4D and E was measured by TEM. Figure 4D shows the TEM images of the MSNs (100 to 150 nm), revealing a clear mesoporous structure. The TEM image of FMSNs (Fig. 4E) also shows a uniform spherical shape even after grafting the A-F complex, and the mesoporous structure was well maintained. Figure 4F was analyzed by low-resolution TEM to observe the PDA coatings; a thin PDA layer is visible around the particles. In addition, the mesoporous structure remained unchanged after PDA coating.

**Fig. 5.**
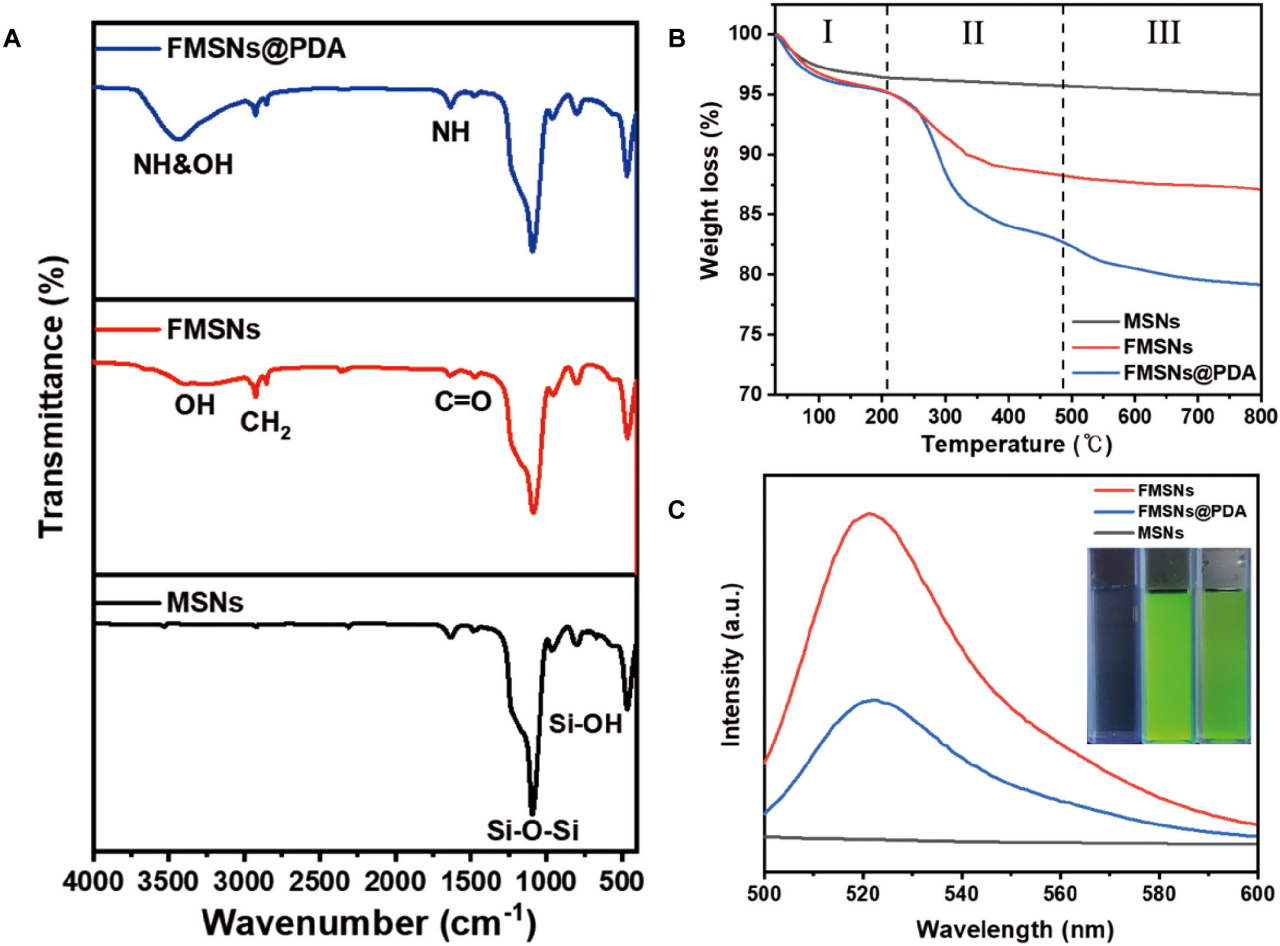
(A) FTIR spectra, (B) TGA and differential scanning calorimetry curve, and (C) photoluminescence spectra of as-prepared samples (MSNs, FMSNs, and FMSNs@PDA).

In the Fourier transform infrared spectroscopy (FTIR; iS50, Thermo Fisher Scientific, Massachusetts, USA) spectrum of the MSNs (Fig. 5A), stretching and asymmetric vibrations of the Si–O–Si bond were observed at 1,092 and 800 cm^−1^, respectively, which are indicative of the characteristic peaks of the silica framework. An asymmetric vibration of Si–OH at 970 cm^−1^ was also identified, which corresponds to the stretching vibration of the silanol group and the presence of adsorbed water. FMSNs exhibited a new peak in the FTIR spectrum corresponding to the A-F complex grafted onto the pore walls. The peaks at 1,480 and 1,640 cm^−1^ indicate the oscillations of the C–O and C═C bonds in the A-F complex. Additionally, the peaks at 2,900 and 2,850 cm^−1^ correspond to the asymmetric and symmetric stretching vibrations of the CH_2_ group, respectively. These peaks indicate successful fluorescent A-F complex grafting onto FMSNs. The FTIR spectrum of FMSNs@PDA showed broad peaks in the OH/NH stretching region ranging from 3,600 to 3,000 cm^−1^. These peaks were more distinct than those of FMSNs, confirming the presence of PDA layer with hydroxyl and amino groups. A new peak at 1,650 cm^−1^ was also identified, which corresponds to NH groups in the aromatic ring. This peak indicates a successful PDA coating on FMSNs.

Figure 5B shows the thermogravimetric analysis (TGA; SDT Q600, Sindosc, Seoul, Korea) of the NPs. The samples were heated to 800 °C under a nitrogen atmosphere. The initial weight loss at approximately 100 °C (stage I) is due to moisture removal. The MSNs maintained their weight after the initial loss. This indicates that CTAB was successfully removed during washing [12]. FMSNs showed an additional weight loss of 12.92 wt % in stage II (~200 °C) due to the weight loss of the grafted A-F complex [13]. FMSNs@PDA exhibited greater weight loss up to 550 °C, followed by a gradual decrease in weight of 20.81 wt % up to 800 °C [14]. Based on the total weight loss (5.04 wt. %) of MSNs, the A-F complex and PDA contents were calculated as 7.88 and 12.93 wt % per particle mass, respectively.

The fluorescence characteristics of the samples were analyzed using photoluminescence spectroscopy, as shown in Fig. 5C. The MSNs exhibited no fluorescence emission peaks, indicating the absence of inherent fluorescence from silica NPs. In contrast, FMSNs and FMSNs@PDA exhibited distinct fluorescence emission peaks at 522 nm.

### Application of various kinetic models

The Korsmeyer–Peppas (K-P) model combines diffusion and relaxation effects to better understand drug release behavior over time. The release kinetics of all the drug systems in this study were analyzed using the K-P model.

$$Q_{t}/Q_{\infty }=k_{R}t^{n}$$

**Fig. 6.**
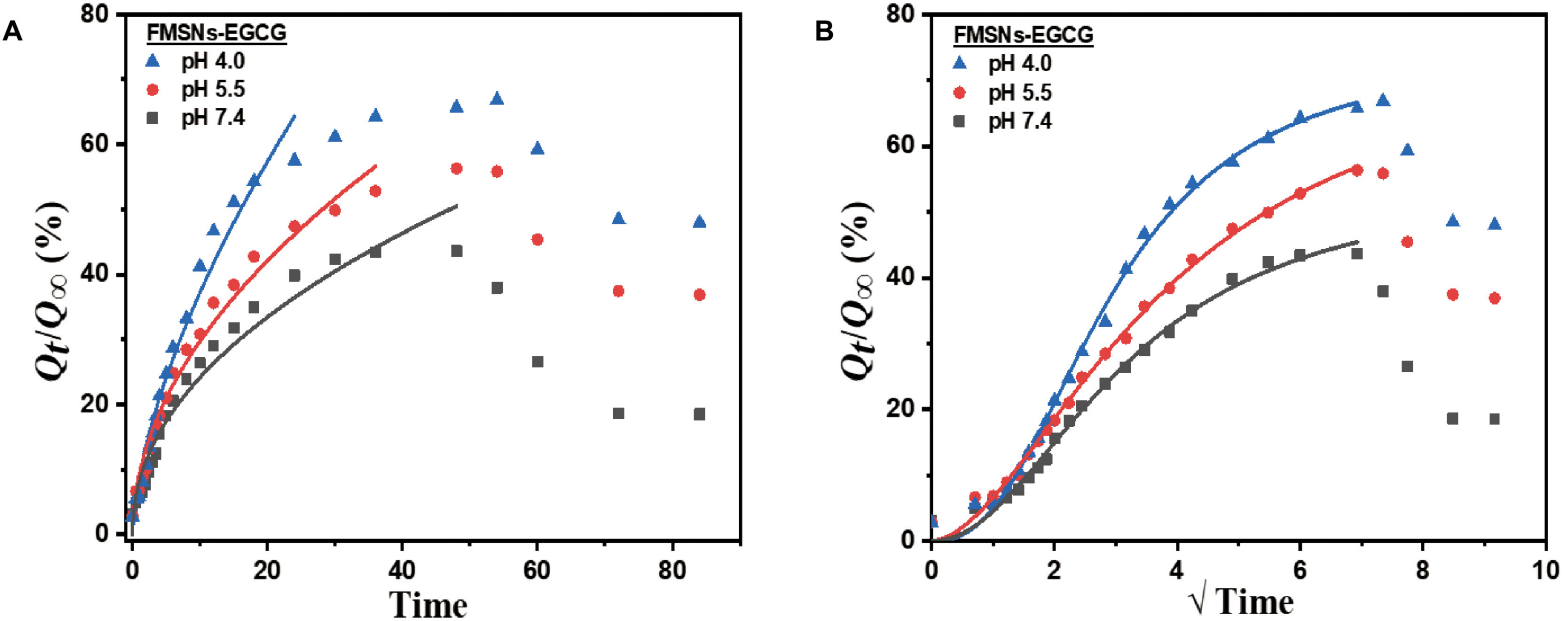
(A) K-P model fits and (B) Hill model fits of drug release EGCG release from FMSNs in PBS at 37 ± 1 °C.

In the equation, *Q_t_*/*Q*_∞_ represents the fraction of drug released at time *t*, *k*_R_ is a constant, and *n* is the release exponent. The release exponent (*n*) determines the release mechanism. The K-P model can be applied to the initial 60% of the cumulative release fraction [15]. However, the release fraction of FMSNs-EGCG was less than 60% due to the concurrent degradation of EGCG during the release process, as modeled by the K-P model (Fig. 6A).

Most release data exhibited *n* values ranging from 0.47 to 0.62, indicating non-Fickian diffusion behavior [16,17]. This suggests that the release kinetics of the FMSN-based nanocarriers do not follow the typical Fickian diffusion. It can be interpreted that as the *n* value increases, the contribution of the release by relaxation also increases. The *k*_R_ and *n* values fitted by the K-P model are summarized in Table S1.

Notably, we observed a sigmoidal profile in the drug release curves, indicating a complex release behavior. To further analyze and understand the release kinetics, we applied the Hill model, also known as the Hill-Langmuir equation. The Hill equation is a mathematical model frequently employed to describe the sigmoidal shape of release behavior [18–20].

$$Q_{t}/Q_{\infty }=\frac{Q_{\max}{*}^{t^{\gamma }}}{{Q_{1/2}}^{n}+t^{\gamma }}$$

In the equation, *Q_t_*/*Q*_∞_ represents the release fraction of drug at time *t*, *Q*_max_ is the maximum amount of drug released from the carriers, γ is the Hill coefficient, and *Q*_1/2_ represents the drug concentration at which half of the maximum drug release occurs. The Hill model analysis was applied to all datasets, as shown in Fig. 6B. The parameter γ exhibited values greater than 1, indicating positive cooperativity between the drug and the delivery system. The fitted parameter values are summarized in Table S1. For FMSNs-EGCG, the *R*^2^ values, mostly exceeding 0.99, indicate reliable and accurate data fitting, supporting the validity of the kinetic analysis.

**Fig. 7.**
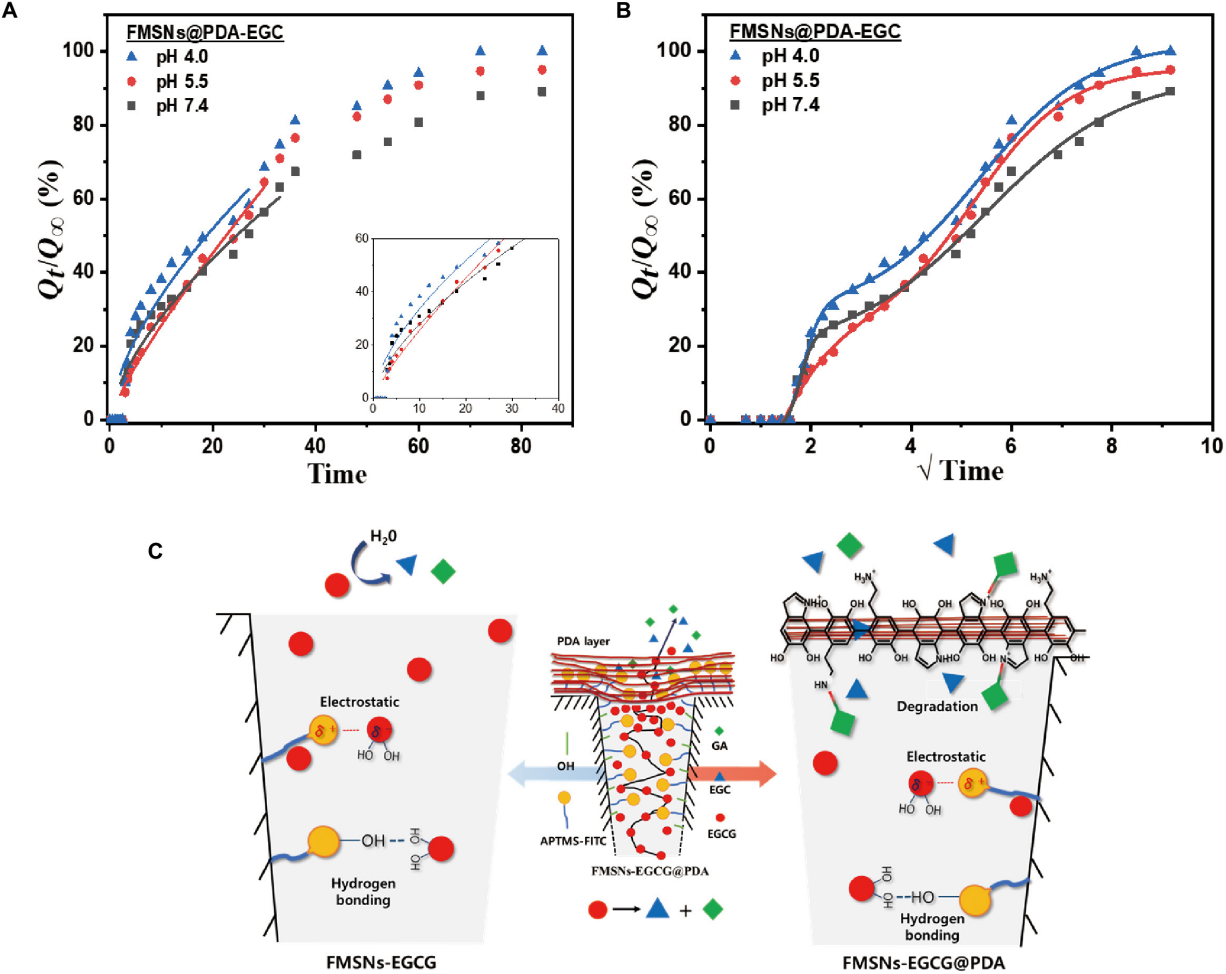
(A) K-P model fit, (B) BiDoseResp model fit of EGC production by FMSNs-EGCG@PDA in PBS at 37 ± 1 °C, and (C) schematic representation of the release mechanism of FMSNs-EGCG@PDA nanocarrier and EGC and GA.

In the case of FMSNs-EGCG@PDA (Fig. 7), the released drug molecules underwent rapid decomposition, leading to an immediate increase in the production rate of EGC, which presumably corresponds to the release rate of EGCG. When examining pristine EGC in an aqueous solution, the absorbance peak of EGC undergoes changes over time, accompanying with a color transition to brown [21]. However, as depicted in Fig. S2, the EGC converted from EGCG showed no discernible peak and no color changes over time. Therefore, we conducted a kinetic analysis of FMSNs-EGCG@PDA by evaluating the cumulative production of EGC, so-called FMSNs@PDA-EGC, which closely mirrors the cumulative release of EGCG. Initially, we employed the K-P model, as shown in Fig. 7A. For FMSNs@PDA-EGC, the kinetic data indicated *n* values ranging from 0.62 to 0.65 and *R*^2^ values ranging from 0.859 to 0.966 (Table S1). Owing to the strong interaction between EGCG and the primary (including secondary) amine groups of PDA, EGCG undergoes rapid degradation as it traverses the PDA coating layer, leading to the prompt generation of EGC in the PDA layer and its subsequent release into the surrounding medium [22–24]. This reaction gives rise to a drug release pattern characterized as anomalous rather than defining non-Fickian behavior.

In the case of FMSNs@PDA-EGC, a 2-stage release pattern was observed under each pH condition so that we applied the BiDoseResp model to analyze the kinetic data (Fig. 7B). This model is mainly used to describe curves with 2 phases of distinct slopes. The production rate of EGC was predicted using a 2-stage BiDoseResp model [18].

$$\begin{array}{c} Q_{t}/Q_{\infty }=\frac{Q_{1}}{Q_{\infty }}+\left(\frac{Q_{2}}{Q_{\infty }}-\frac{Q_{1}}{Q_{\infty }}\right)\\ \left[\frac{A}{1+10^{\log}\left({\left(t_{1}-t\right)}^{0.5}\right)h1}+\frac{1-A}{1+10^{\log}\left({\left(t_{2}-t\right)}^{0.5}\right)h2}\right] \end{array}$$

Here, we defined *Q_t_*/*Q*_∞_ as the cumulative fraction of EGC at time *t*; *Q*_1_/*Q*_∞_ and *Q*_2_/*Q*_∞_ are the EGC fraction in the solution at the startup and shutdown release processes, respectively; *h*_1_ and *h*_2_ are the Hill coefficients controlling the slope of each step; *t*_1_ and *t*_2_ are the release processing times of the first and second stages, respectively.

All fitted data exhibited *R*^2^ values greater than 0.99. A slope analysis was performed at the 2 stages after initial delay, revealing the following results: The *h*_1_ value was 2.2 at pH 4.0, 0.7 at pH 5.5, and 3.3 at pH 7.4, demonstrating distinct differences at each pH level. At pH 4.0, 5.5, and 7.4, *h*_2_ values were 0.390, 0.463, and 0.313, respectively, showing lower values than unity and indicating a more stable response than *h*_1_.

The nanocarriers exhibit distinct release behaviors depending on the absence or presence of the PDA coating layer. As shown in Fig. 7C, the initial release of EGCG is delayed owing to electrostatic forces and hydrogen bonding with the A-F complex. Over time, the deprotonation of the A-F complex leads to a reduction in binding [25]. Owing to osmotic pressure, a larger amount of drug is released in the early stages compared to the later stages. Throughout this phase, the released EGCG in the solution decomposes via hydrolysis. In contrast, when EGCG interacts with the PDA layer during its transmission, it facilitates a decomposition reaction, resulting in a relatively fast release in the early to middle stages of the drug release process. This accelerated release is likely because smaller molecules are released more rapidly upon reacting with PDA. The degradation of EGCG in the release medium persist at all pH levels, and its interaction with PDA further accelerated the production of EGC.

### Antioxidant activity based on the release behavior of EGCG

**Fig. 8.**
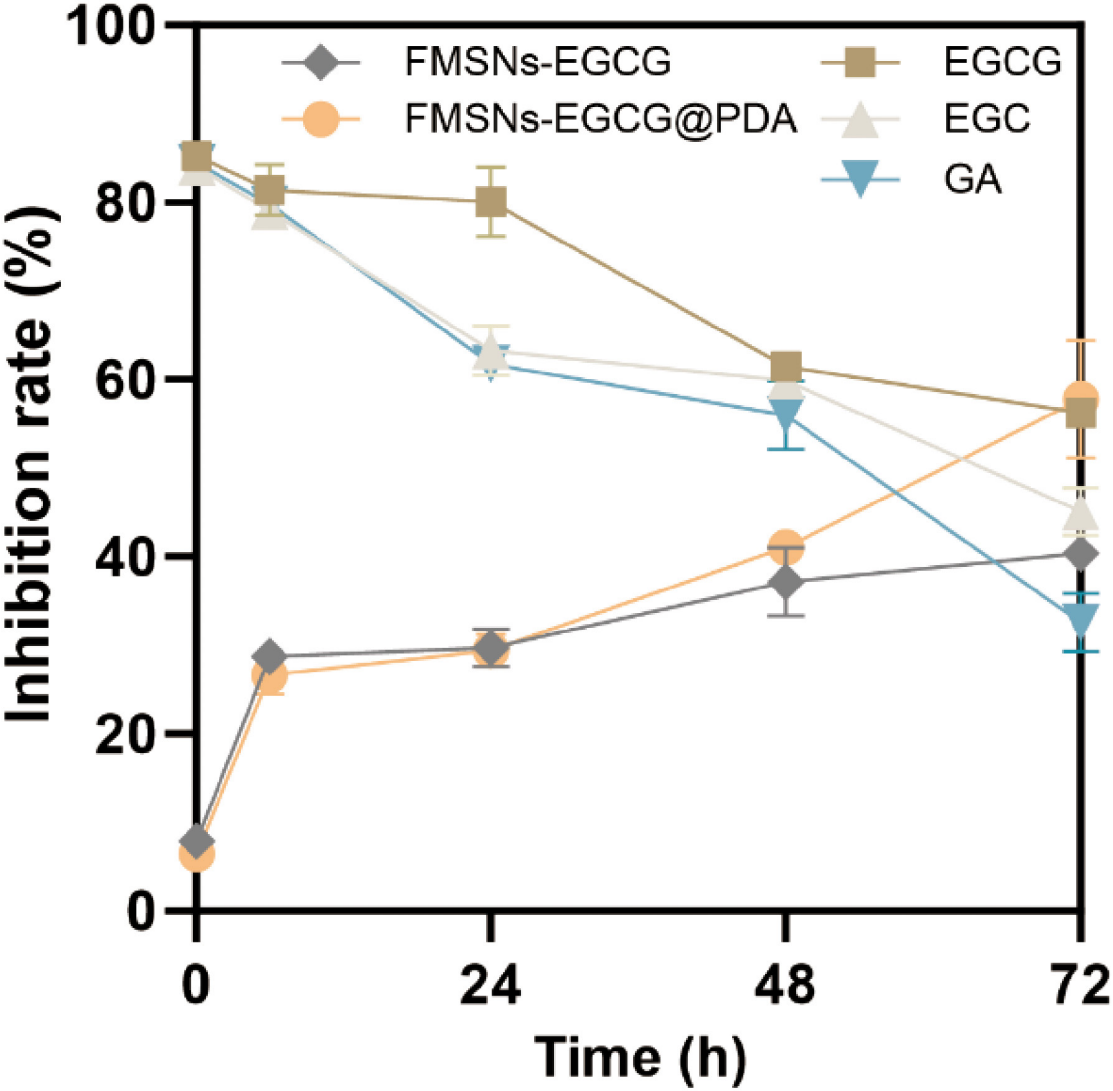
Radical scavenging activity of EGCG, EGC, GA, FMSNs-EGCG, and FMSNs-EGCG@PDA in the DPPH assay at 0, 6, 24, 48, and 72 h. Each value represents the mean ± SD (*n* = 3).

EGCG was used at the highest nontoxic concentration of 25 μM, and its reaction with DPPH solution was studied at different time points (0, 6, 24, 48, and 72 h) (Fig. 8). Examination of the graph showed that EGCG maintained its highest antioxidant efficacy during the first 24 h. However, the drug underwent rapid oxidation by radicals over time, resulting in decreased DPPH inhibition, as evidenced by a 56.2 ± 1.1% reduction at 72 h.

The DPPH assay of EGC and GA, the degradation products of EGCG, was performed to evaluate radical scavenging activity after degradation. EGC and GA were tested at the same concentration as EGCG, 25 μM. EGC and GA showed an inhibition rate of over 80% at the initial time point, which gradually decreased. EGC showed a slightly lower antioxidant efficacy than EGCG at 72 h, reaching 45.1 ± 2.2%. In contrast, the radical inhibition rate of GA decreased sharply at 48 h and reached 32.6 ± 2.7% at 72 h.

The EGCG encapsulated in NPs, FMSNs-EGCG and FMSNs-EGCG@PDA, showed a gradual increase in radical scavenging activity, indicating that the drug was released gradually and maintained its efficacy for up to 72 h. At 72 h, the inhibition rates were 40.3 ± 0.6% and 57.8 ± 5.4%, respectively. FMSNs-EGCG@PDA exhibited a significantly higher radical inhibition. This was attributed to the long-term drug release and radical scavenging ability of PDA. PDA itself possesses radical scavenging abilities, as previously reported [26].

### Assessment of cell viability upon treatment with FMSNs

**Fig. 9.**
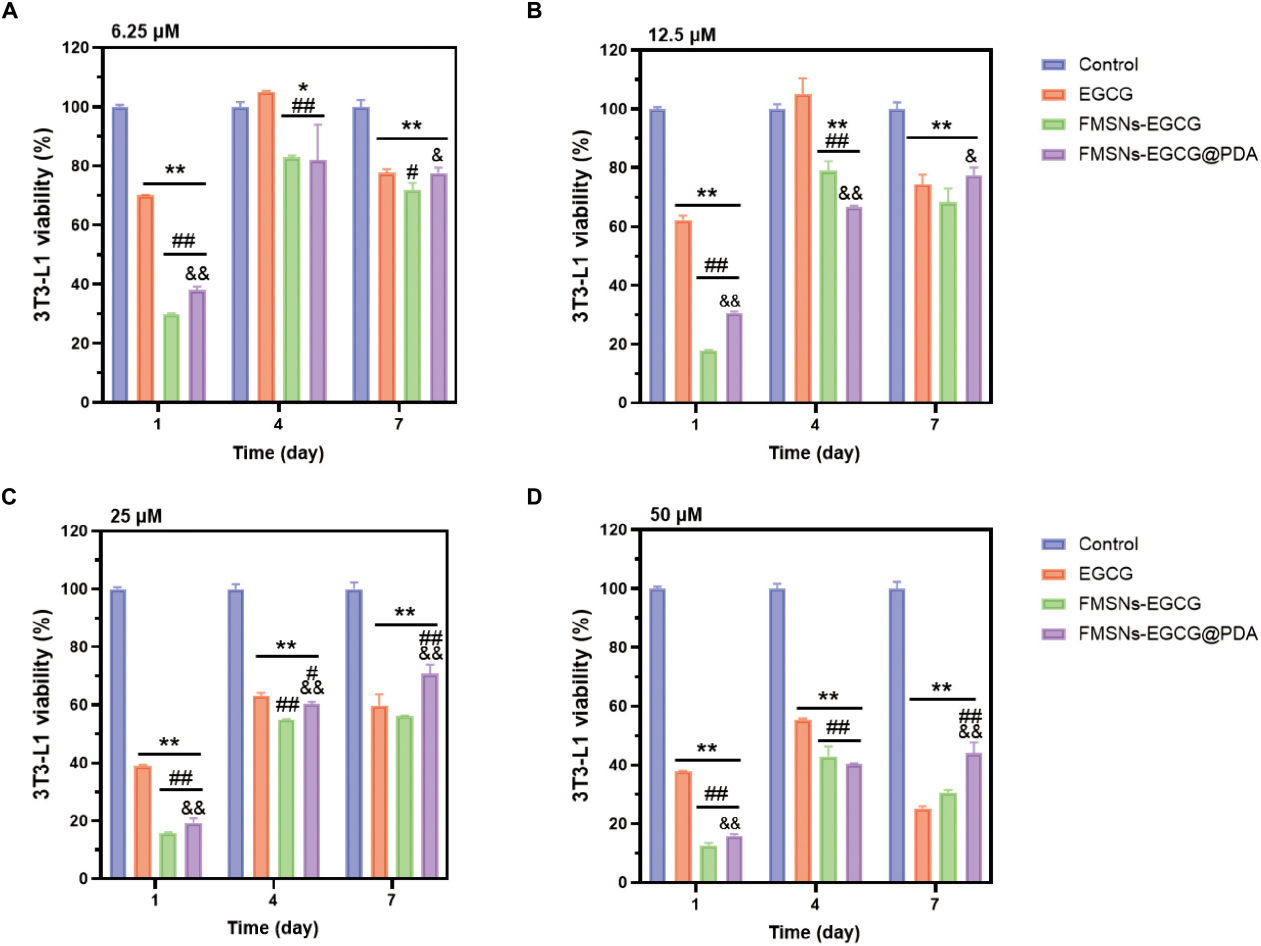
3T3-L1 viability by EGCG, FMSNs-EGCG, and FMSNs-EGCG@PDA at (A) 6.25 μM, (B) 12.5 μM, (C) 25 μM, and (D) 50 μM in the MTT assay on days 1, 4, and 7. Each value is the mean ± SD (*n* = 3). Significant differences are indicated: *P* < 0.05 (*, #, &) and *P* < 0.01(**, ##, &&). The symbols *, #, and & indicate a difference from control, EGCG, and FMSNs-EGCG for each concentration.

EGCG, FMSNs-EGCG, and FMSNs-EGCG@PDA, each treated at different concentrations, were subjected to MTT assays to assess their cytotoxicity on days 1, 4, and 7. In the untreated control group, the cell population steadily grew over time (Fig. 9).

All EGCG-treated groups showed a significant inhibition of cell proliferation. Higher concentrations of EGCG resulted in a more pronounced inhibitory effect on cell proliferation, with a significant decrease in cell viability at 50 μM between days 4 and 7.

FMSNs-EGCG and FMSNs-EGCG@PDA showed lower 3T3-L1 viability than in the control group due to the release of EGCG. On day 1, the EGCG group showed higher cell viability than the FMSNs-EGCG@PDA group at all concentrations. However, by day 7, the cell viability of the FMSNs-EGCG@PDA group exceeded that of the EGCG group, indicating a significant improvement in the performance of FMSNs-EGCG@PDA over 7 days. The cell viability of FMSNs-EGCG@PDA consistently exceeded that of FMSNs-EGCG, confirming the beneficial effects of the PDA coating. This effect resulted from the appropriately delayed release of EGCG while maintaining the desired concentration.

### Effect of EGCG released from FMSNs on the inhibition of 3T3-L1 adipogenesis

The differentiation of 3T3-L1 preadipocytes into mature adipocytes involves fat accumulation and is a central process in obesity. Effective control of adipogenesis is critical for obesity prevention. In the present study, we investigated the efficacy of the antioxidant EGCG in inhibiting adipocyte differentiation via cellular uptake. After 8 days of differentiation induction with specific drugs, Oil Red O staining was used to evaluate the extent of adipocyte formation.

**Fig. 10.**
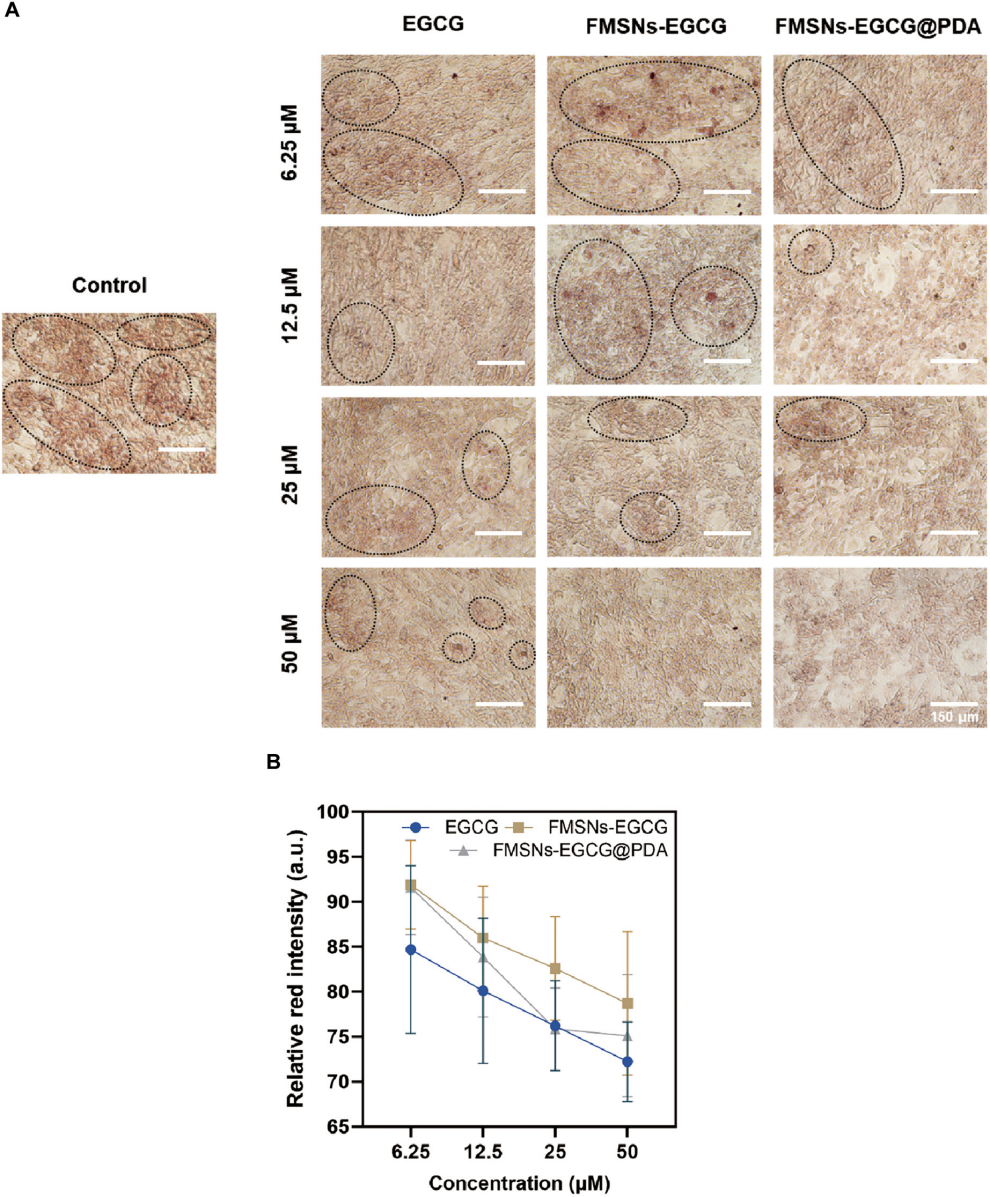
Effects of EGCG on the inhibition of 3T3-L1 adipogenesis. (A) Optical image of 3T3-L1 adipocytes treated with different concentrations of EGCG, FMSNs-EGCG, and FMSNs-EGCG@PDA. Lipids were stained with Oil Red O on day 8. The dotted lines indicate the area of high-density lipid droplets. Scale bars, 150 μm. (B) Relative intensities measured with ImageJ.

The effects of EGCG concentration and particle application were investigated using Oil Red O staining results (Fig. 10). The 3T3-L1 control group, which was not treated with EGCG, showed high red intensity over the entire area. In all EGCG-treated groups, both red intensity and area decreased compared to the control group. When the concentration effect was analyzed, a significant reduction in Oil Red O staining area and intensity was observed with increasing EGCG concentration from 6.25 to 50 μM, highlighting the adipogenesis inhibitory effect of EGCG. No significant differences were observed between the FMSNs-EGCG group, the FMSNs-EGCG@PDA group, and the EGCG group without particle application. For quantitative comparison, the red intensity of the obtained images was analyzed using ImageJ and normalized to the control group (Fig. 10B). As observed in the images, there was an inverse correlation between increasing concentration and decreasing red intensity in all groups. The EGCG group showed the lowest intensity, and the presence of the PDA coating showed a slightly more pronounced effect on fat inhibition compared to the noncoated EGCG group.

## Discussion

The hydrodynamic size and zeta potential measurements provide comprehensive insights into the physicochemical properties of the surface-modified nanocarriers. Initially, the internal grafting of fluorescent conjugates increased the NP size from 150 to 155 nm. Subsequently, an additional external PDA coating further increased the size from 155 to 164 nm. This stepwise increase in size confirms the successful surface modification of the NPs.

The zeta potential analysis reveals critical information about the surface charge and colloidal stability of the nanocarriers. FMSNs exhibited an increase in zeta potential to a more positive value, attributed to the grafting of the positively charged A-F complex [27]. In contrast, the zeta potential of FMSNs@PDA exhibited a significant decrease, reaching −33.98 ± 4.21 mV, indicative of a high degree of colloidal stability. The decrease is likely due to the catechol groups in PDA, which predominantly exist in the quinone form at neutral pH [28]. Overall, the hydrodynamic size and zeta potential measurements confirm the successful surface modification and highlight the enhanced colloidal stability of the nanocarriers.

The surface area and pore size analysis using BET adsorption isotherm and BJH pore distribution further elucidate the structural integrity of the modified nanocarriers. The isotherm pattern, similar to an IV-type isotherm, is typical for porous materials with a constant cross-section, indicating well-defined porous structures. The PDA coating, although it may have partially filled the pores and slightly reduced the pore size, did not alter the overall mesoporous nature of the NPs [29]. The BJH analysis showed that all pores remained within the mesoporous range of 2 to 50 nm. These findings suggest that the original mesoporous structure remained intact despite the surface modifications, including A-F complex grafting and PDA coating.

The morphological studies using SEM and TEM confirmed the uniform spherical shape and consistent size of the NPs, regardless of the surface modifications. TEM images revealed a clear mesoporous structure in MSNs and FMSNs, with FMSNs@PDA showing a thin PDA layer around the particles. Importantly, the mesoporous structure remained unchanged after the PDA coating, which is critical for maintaining the functional properties of these nanocarriers.

FTIR analysis provided direct evidence of the chemical modifications on the nanocarriers. The characteristic peaks of the silica framework were observed in the MSNs, while new peaks corresponding to the A-F complex appeared in FMSNs. The FMSNs@PDA spectrum exhibited broad peaks in the OH/NH stretching region and a new peak at 1,650 cm^−1^, confirming the presence of the PDA layer with hydroxyl and amino groups. These results affirm the successful grafting of the A-F complex and the subsequent PDA coating on the nanocarriers.

TGA analysis revealed the thermal stability and composition of the modified nanocarriers. The MSNs showed minimal weight loss, indicating the effective removal of CTAB during washing. FMSNs exhibited additional weight loss due to the A-F complex, while FMSNs@PDA showed greater weight loss up to 550 °C, attributed to the presence of PDA. The quantitative analysis of the weight loss provided insights into the content of the A-F complex and PDA in the nanocarriers.

The fluorescence characteristics of the nanocarriers were analyzed using photoluminescence spectroscopy. FMSNs and FMSNs@PDA exhibited distinct fluorescence emission peaks at 522 nm, attributed to the fluorescent A-F complex and its interaction with the silica NPs. The fluorescence intensity was higher in FMSNs compared to FMSNs@PDA, possibly due to the additional PDA layer, which could absorb or hinder part of the fluorescence. The intense green fluorescence emitted by FMSNs and the darker green fluorescence from FMSNs@PDA demonstrate the fluorescence capabilities of the FITC-conjugated nanocarriers. This feature enables real-time fluorescence tracking of delivery routes and validation of successful transportation to the target area, enhancing the functionality of these nanocarriers for biomedical applications.

The primary emphasis was on investigating release kinetics of EGCG from mesoporous nanocarriers to understand the behavior of drug delivery systems and to improve their performance through optimization. Figure S3 presents the time-dependent absorbance of EGCG released from the nanocarriers in PBS at various pH values (pH 7.4, 5.5, and 4.0). As shown in Fig. S3A, the release profile of FMSNs-EGCG indicated that the release behavior of EGCG was maintained for approximately 50 h. Following this period, a decrease in the absorbance of EGCG was observed, possibly indicating the degradation of EGCG after its release into PBS [30].

Figure S3B shows the release profiles of FMSNs-EGCG@PDA, in which FMSNs-EGCG @PDA exhibits an initial increase in absorbance followed by a rapid decrease after 5 h, showing a chaotic fluctuating variation in absorbance. This suggests that the PDA coating on the nanocarrier influences the release behavior of EGCG, potentially leading to an increased release rate. These findings offer valuable insights into the release kinetics of EGCG from mesoporous nanocarriers. The prolonged release observed in FMSNs-EGCG and the faster release of FMSNs-EGCG@PDA suggest that the selection of nanocarriers and any additional coatings or modifications can greatly influence the drug release behavior.

Figure S4 indicates that there is no clear effect on absorbance enhancement by PDA coating, in contrast with the distinct absorbance peak of the EGCG drug. The absorption peaks of NPs (FMSNs, FMSNs@PDA) and EGCG appear at 520 and 274 nm, respectively. The 520-nm peak is attributed to the A-F complex grafted on the MSNs, referred to as FMSNs. Upon PDA coating on FMSNs (referred to as FMSNs@PDA), it was observed that the peak intensity slightly decreased probably due to the presence of the PDA coating layer on FMSNs. Notably, in measuring drug release amount via absorbance change, particles were isolated through centrifugation to exclusively measure EGCG peak only. Furthermore, the potential for rapid drug release attributed to the light absorption by PDA was effectively eliminated by conducting the in vitro release test in a dark environment.

The release of EGCG from the mesoporous nanocarriers was attributed to the hydrolytic degradation of EGCG in PBS during the release test [31]. It is generally known that EGCG tends to degrade faster under neutral pH conditions, yielding EGC and GA by-products [9,30].

Figure S5A shows the time-dependent absorbance of EGC in the release medium of FMSNs-EGCG at each pH level, which is the degradation product of EGCG. The EGC amount in the solution was measured to assess the release behavior of EGCG that is easily converted into EGC via hydrolysis. The lag time of the FMSNs-EGCG nanocarriers was examined, and EGC was produced after 1.5 h at pH 7.4, 3.5 h at pH 5.5, and 5 h at pH 4.0, clearly indicating that EGCG is more rapidly degraded into EGC at the higher pH level. To calculate the total amount of drug released from the nanocarrier, the absorbance of the EGC and EGCG components was analyzed in the final release medium. At pH 7.4, EGCG and EGC accounted for 10% and 64% of the total, respectively, resulting in a final release of 74% (96.94 mg/g). At pH 5.5, the ratios of EGCG and EGC were 21% and 58%, respectively, resulting in a final release of 79% (103.49 mg/g). Similarly, at pH 4.0, the proportions were 30% and 52%, respectively, resulting in the final release of 82% (107.42 mg/g).

Figure S5B shows the time-dependent absorbance of EGC in the release medium of FMSNs-EGCG@PDA. The lag time for FMSNs-EGCG@PDA nanocarriers indicated consistent EGC production after 1.5 h across all pH levels. The drug release percentages from FMSNs-EGCG@PDA were approximately 84% (70.98 mg/g) at pH 7.4, 91% (76.90 mg/g) at pH 5.5, and 95% (80.28 mg/g) at pH 4.0. Notably, EGCG was not detected in the final release medium in the presence of PDA-coated nanocarriers.

According to the mechanistic interpretation shown in Fig. S6A, hydrolysis of EGCG can occur at the ester linkages, generating EGC and GA by-products. When PDA is employed as an outer coating for the nanocarrier, both amine and catechol groups are present on the surface of FMSNs@PDA [32]. The zeta potential of FMSNs@PDA exhibits an increase in acidic conditions, which is attributed to the amine groups in PDA, while it decreases under neutral or alkaline conditions owing to the prevalence of multiple catechol groups in PDA [28], as summarized in Table S2. PDA is highly effective for immobilizing biomolecules and readily forms strong bonds with EGCG. This binding promotes the release of EGCG by facilitating its degradation via interactions with the primary and secondary amine groups of PDA (Fig. S6B) [33,34]. As illustrated in Fig. S6C, the amide bond undergoes hydrolysis facilitated by H^+^ and OH^−^ ions, leading to the separation of PDA and GA by breaking the bond between them.

The application of the K-P model revealed that the release kinetics of the FMSN-based nanocarriers follow non-Fickian diffusion behavior, suggesting that both diffusion and relaxation effects contribute to the drug release. The lower than 60% release fraction for FMSNs-EGCG highlights the concurrent degradation of EGCG, emphasizing the need to account for degradation in kinetic modeling. The Hill model analysis showed sigmoidal release profiles with positive cooperativity, indicating a complex interaction between EGCG and the delivery system. The high *R*^2^ values validate the kinetic analysis and suggest reliable fitting of the experimental data.

In the interpretation of the sigmoidal release behavior, it is important to consider that EGCG is released and decomposed simultaneously. This led to a time-evolution decrease in the amount of EGCG and a simultaneous increase in the amount of its decomposition product, EGC, over time. The delivery system exhibited a low initial emission due to strong intermolecular interactions between the positively charged FMSNs and the negatively charged EGCG drug [35,36]. However, the deprotonation of the A-F complex over time reduced electrostatic attraction and hydrogen bonding, leading to increased release rates. The initially low release rate transitioned into varying rates in subsequent steps, thereby producing an S-shaped release profile. It had the largest γ value at pH 4.0, mainly because of the sustained release of EGCG (which is more stable in acidic conditions) and the relatively slower generation of EGC. At pH 7.4, a minimal amount of EGCG remained in the release medium because of its rapid conversion into EGC by hydrolysis, resulting in an S-shaped appearance. However, at pH 5.5, the amounts of EGCG released and EGC produced in the solution were comparable, resulting in a less pronounced S shape. As a result, pH 5.5 fits a lower γ value than pH 7.4.

For FMSNs-EGCG@PDA, the kinetic analysis indicated anomalous behavior due to strong interactions between EGCG and PDA’s amine groups, leading to rapid degradation and release of EGC. The BiDoseResp model effectively captured the 2-stage release pattern, with initial rapid release followed by a more stable phase. The first stage involves the production of EGC through both the internal transmission of EGCG by reacting with PDA layer and the hydrolytic decomposition of EGCG in the solution. The second stage involves the production of EGC only through the transmission of EGCG reaction with PDA. When comparing the slopes of the first and second stages, the *h*_1_ value (first stage) was higher than the *h*_2_ value (second stage). Under acidic conditions, the amino group of PDA undergoes deprotonation, leading to less effective closure of the pores [37]. At pH 4.0, compared with other pH levels, the EGCG and degradation products are easily escaped through the loosened PDA at acidic pH condition, producing a high value of *h*_1_. At pH 5.5, the *h*_1_ value was less than 1, indicating that the release rate is comparable to the production rate of EGC in the solution. Conversely, at pH 7.4, the *h*_1_ value was the highest, while the *h*_2_ value was the lowest. This suggests that at neutral pH, EGC is rapidly produced at the first stage, but the final amount of EGC is lower than those obtained at acidic pH levels. All the fitted parameters are summarized in Table S1.

The distinct release behaviors observed with and without PDA coating underscore the importance of surface modifications in tailoring drug release profiles. The initial delay in EGCG release due to electrostatic forces and hydrogen bonding transitions to a faster release phase as binding forces reduce over time. The interaction with PDA accelerates EGCG degradation, enhancing release rates in the early to middle stages. These findings suggest that precise control of surface modifications and environmental conditions can optimize drug delivery systems for specific therapeutic needs.

In the initial assessment of antioxidant capacity, EGCG, EGC, and GA exhibited substantial antioxidant potential. However, progressive divergence in the ability to sustain this antioxidant activity became apparent over time. Typically, the source of antioxidant efficacy lies within the phenolic structure, and it can be inferred that a greater abundance of phenolic structures corresponds to an increased antioxidant effect [38]. The number of phenolic structures in EGCG, EGC, and GA followed the order EGCG > EGC > GA, reflecting the pattern of radical inhibition at 72 h. The lack of significant differences in the effects of EGCG, EGC, and GA could be attributed to their concentrations exceeding the range that could be effectively evaluated using the DPPH assay. However, the differences in the antioxidant effects of these compounds became more pronounced over time.

The administration of EGCG without particle encapsulation resulted in a strong initial antioxidant capacity that gradually decreased over time. However, when EGCG was encapsulated in the particles, its antioxidant potential showed gradual time-dependent enhancement. Particle encapsulation prolonged the duration of the antioxidant effect, and when coupled with PDA coating, it was evident that the antioxidant effect was maintained for a longer period.

The cell viability results indicate that higher concentrations of EGCG significantly inhibit cell proliferation, particularly evident between days 4 and 7. This highlights the need to carefully manage EGCG concentrations to avoid cytotoxic effects. Encapsulation of EGCG in FMSNs and FMSNs-EGCG@PDA mitigated these effects, with the PDA coating providing additional benefits. On day 1, the direct treatment with EGCG showed higher cell viability, but by day 7, FMSNs-EGCG@PDA demonstrated superior performance. This improvement is attributed to the sustained release and better management of EGCG concentrations over time, which is crucial for maintaining cell viability and proliferation.

The enhanced performance of FMSNs-EGCG@PDA is further supported by improved cell adhesion properties of PDA, leading to increased cellular uptake of EGCG [39]. After culturing 3T3-L1 cells with FMSNs and FMSNs@PDA, particle uptake was examined by fluorescence microscopy (Fig. S7). In the early stages of cultivation, strong fluorescence was observed in distinct areas due to particle aggregation. However, as the incubation time increased, the particles were taken up by the cells and distributed evenly throughout the cells, a common observation. FMSNs@PDA showed a more uniform distribution of fluorescence over a larger area compared to FMSNs. In addition, on day 4, it was evident that the total distribution area of fluorescence was larger for FMSNs@PDA compared to FMSNs. This suggests an enhanced drug delivery effect due to increased cellular uptake.

The results demonstrate that EGCG effectively inhibits adipogenesis in 3T3-L1 preadipocytes, with increasing concentrations leading to more significant reductions in fat accumulation. This is evidenced by the decreased red intensity and area in Oil Red O staining, confirming EGCG’s adipogenesis inhibitory effect. Variations in the distribution and staining intensity of 3T3-L1 were observed based on microscopy capture locations, resulting in significant variations in the quantification analysis performed on image-based data and a lack of statistical significance. However, despite these challenges, trends with increasing concentration and application of EGCG to FMSNs showed consistent effects.

While our experiments have shown that EGCG is the most promising candidate, practical applications face some limitations. The poor water solubility of EGCG, around 10 μM, results in a very low applicable concentration. In this study, EGCG was dissolved in DMSO and then diluted for use. In contrast, when applied to FMSNs, EGCG loading can be achieved without organic solvents, providing an advantage for practical use at higher concentrations. Another consideration is long-term use. This study primarily focused on investigating the adipogenesis inhibition of EGCG delivered by PDA-coated FMSNs using in vitro experiments with 3T3-L1, emphasizing sustained release and potential adipogenesis inhibition. The nature of cells gradually changes after differentiation of 3T3-L1, making long-term cultivation challenging. Therefore, the adipogenesis inhibition effect was only measured once on day 8 in this experiment. The limitation of not addressing the confirmation of effects through repeated and long-term applications, possibly through animal experiments, remains to be explored.

Research on the adipogenic inhibitory effects and mechanisms of EGCG through cell experiments based on 3T3-L1 cells has been relatively well explored, but in vivo experiments are limited. Application of EGCG to mice has shown effects such as weight loss and reduction of abdominal fat [27,28]. However, existing animal studies involved daily oral administration of EGCG for more than 8 weeks, and EGCG is considered a dietary supplement rather than a drug. In our study, we consider EGCG as a drug and incorporate it into a controlled-release delivery system, which we expect to be more effective than conventional methods. Our focus in this research is to validate the behavior of EGCG loaded on FMSNs and its in vitro efficacy, and there are limitations in ensuring clinical efficacy. We anticipate that future studies will involve direct, single administration of EGCG-loaded FMSNs into adipose tissue and verify clinical efficacy by observing changes in adipose tissue.

In this study, FMSN acted as an effective drug delivery system, increasing drug stability and providing sustained release over time. The explosive EGCG degradation during PDA coating highlights the complexity of drug–particle interactions and emphasizes the importance of comprehensive research in this field. We used sophisticated mathematical models, including the K-P, Hill, and BiDoseResp, to describe drug release and production mechanisms. These models allowed the prediction and description of the continuous release profile of the drug and revealed its underlying kinetics. The therapeutic potential was evaluated using antioxidant and adipocyte differentiation inhibition assays, demonstrating its clinical applicability. We demonstrated significant free-radical scavenging and effective inhibition of adipocytes, highlighting the potential clinical relevance of our approach. This study revealed the synergistic enhancement of the antioxidant effects of EGCG, with potential applications in the treatment of oxidative stress, obesity, and antioxidant therapy. These findings provide valuable insights for developing innovative therapeutic strategies for obesity suppression, oxidative stress management, and antioxidant therapies.

## Data Availability

The datasets used and/or analyzed during the current study are available from the corresponding author on reasonable request.
