# Supplementary material for: Optimized Epigallocatechin Gallate Delivery and Adipogenesis Inhibition through Fluorescent Mesoporous Nanocarriers
Source: Biomater Res. 2024 Jul 16;28:0053. doi: 10.34133/bmr.0053 (PMC11249910; doi:10.34133/bmr.0053)
Supplement: Supplementary 1 — Supplementary Text Figs. S1 to S7 Tables S1 and S2 [file bmr.0053.f1.docx]

**Optimized Epigallocatechin Gallate Delivery and Adipogenesis Inhibition through Fluorescent Mesoporous Nanocarriers**

Taelin Kim^1^, A Yeon Cho^1^, Sang Wha Lee*, Hyun Jong Lee*

*Department of Chemical and Biological Engineering, Gachon University, 1342 Seongnam-daero, Seongnam-si, Gyeonggi-do 13120, Republic of Korea***E-mail: hjlee2@gachon.ac.kr, lswha@gachon.ac.kr*

^1^These authors contributed equally.

**Instrument of analysis**

The surface morphology of the samples was characterized by Scanning Electron Microscope (SEM, S-4700, Hitachi). The distinct nanostructure morphology of the samples was characterized by Transmission Electron Microscope (TEM, H7600, Hitachi) at an accelerating voltage of 80 kV. Zeta potential and particle size of the samples were measured by Electrophoretic Light Scattering method (ELSZ-2000, Otsuka). The functional groups of the samples were characterized by Fourier transform infrared spectroscopy (FTIR, Vertex 70, Bruker) in the range of 4000 to 500 cm^-1^. Photoluminescence spectroscopy (PL) was used to measure the fluorescence of the samples under an excitation wavelength of 470 nm. UV-vis spectroscopy (HP 8453, Agilent Technologies) was used to characterize the optical properties of as-prepared samples. The thermal properties of the samples were analyzed using Thermo Gravimetric Analysis (TGA, SDT Q600).


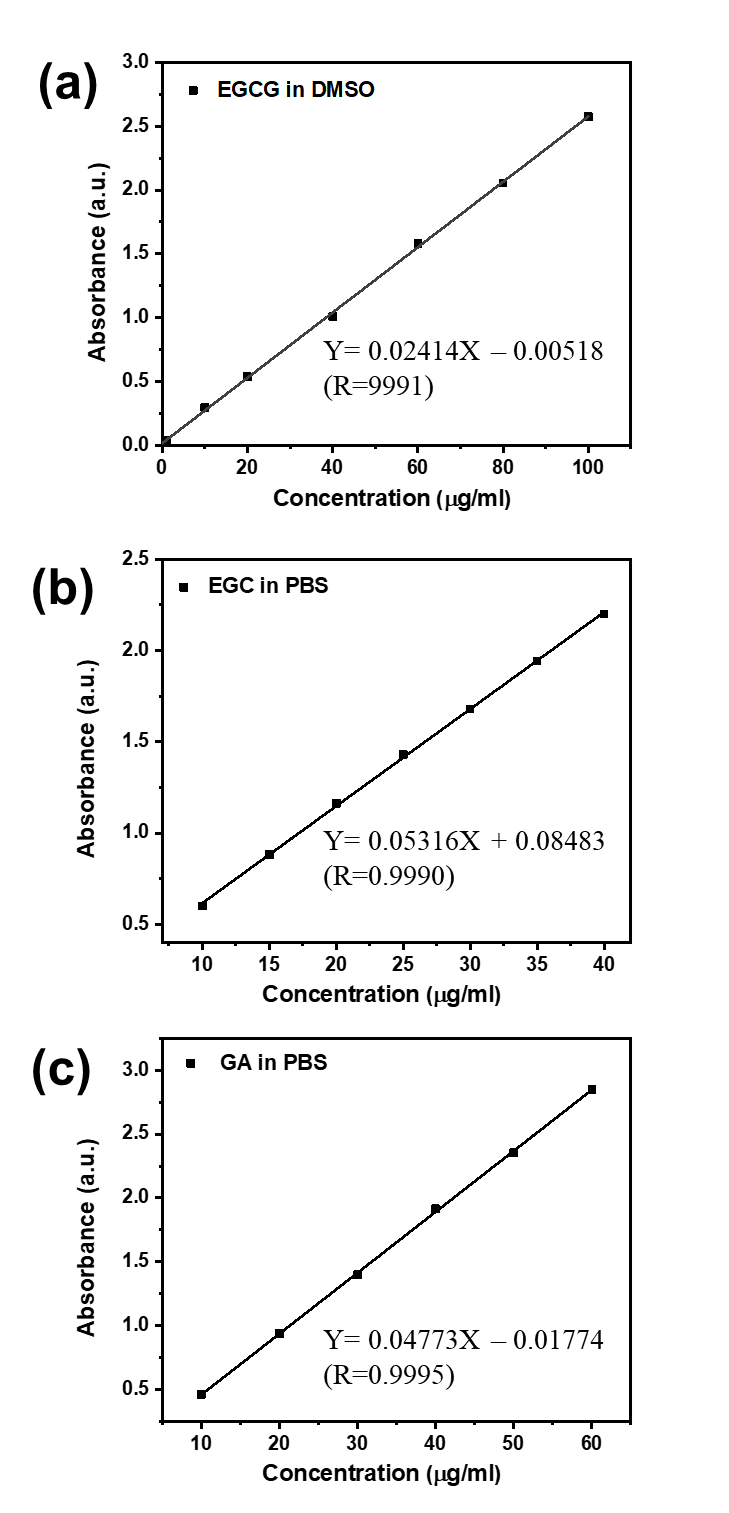


**Fig. S1.** Standard curve of (a) EGCG in DMSO, (b) EGC in PBS pH 7.4, and (c) GA in PBS pH 7.4

**
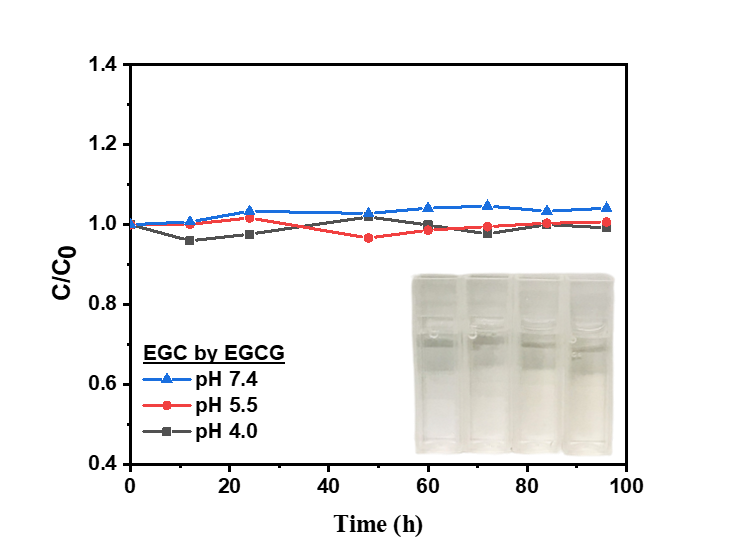
**

**Fig. S2.** Time-dependent absorbance changes of EGC generated by the degradation of EGCG in PBS.

**
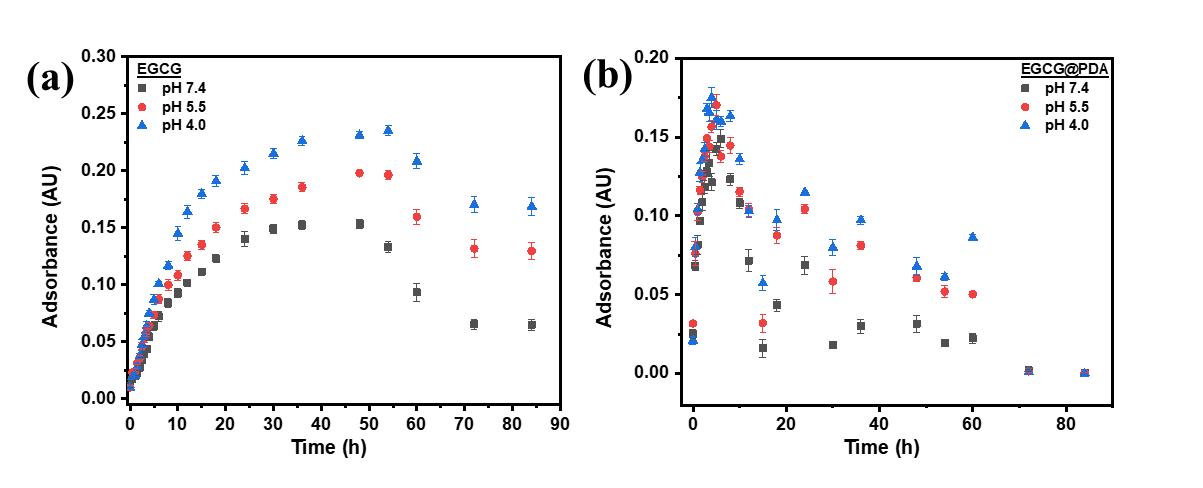
**

**Fig. S3.** Release profiles of EGCG absorbance from FMSNs and FMSNs@PDA in PBS at 37 ± 1 ℃.

**
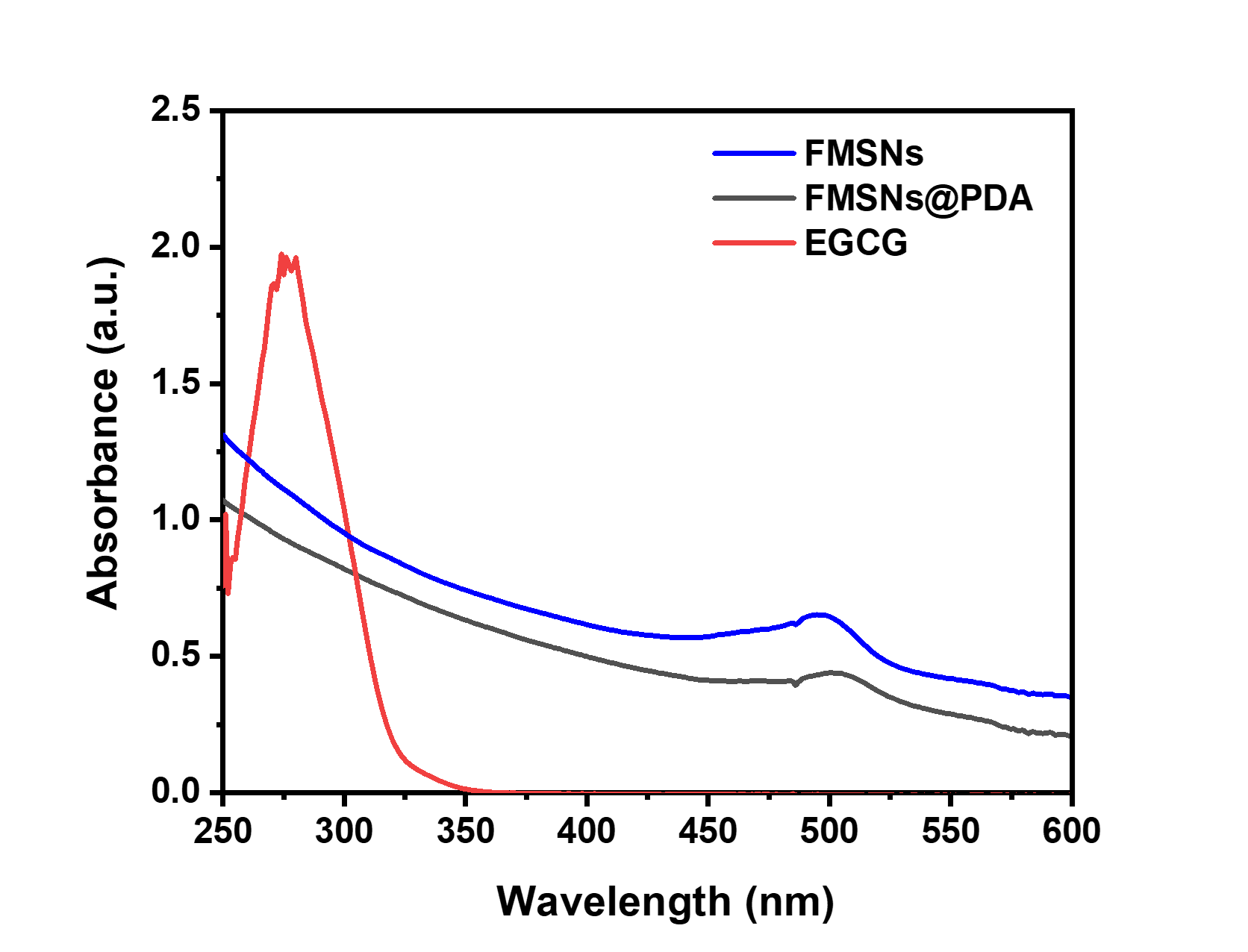
**

**Fig. S4.** UV-vis spectra of the samples (FMSNs, FMSNs@PDA) and EGCG in PBS.

**
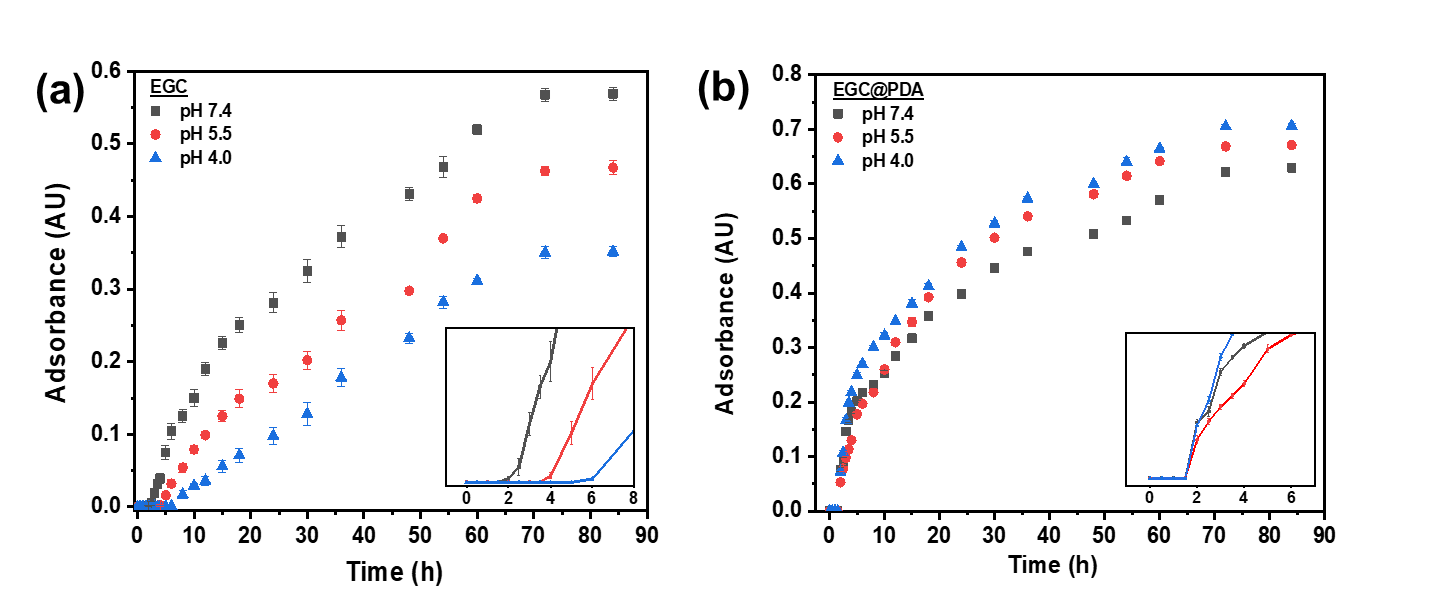
**

**Fig. S5.** Release profiles of EGC absorbance from FMSNs and FMSNs@PDA in PBS at 37 ± 1 ℃.

**
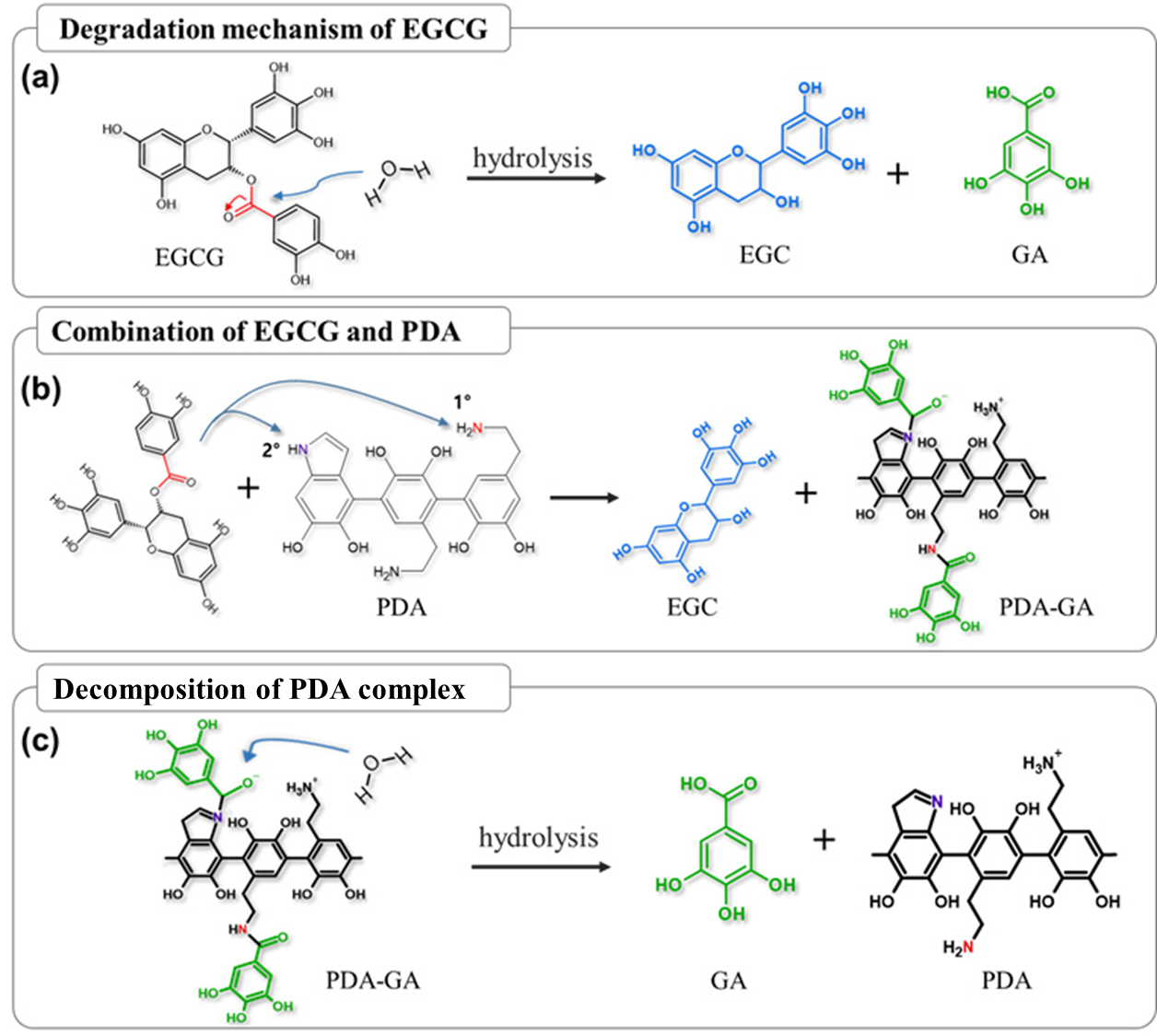
**

**Fig. S6**. (a) Degradation mechanism of EGCG, (b) Combination of EGCG and PDA, and (c) Decomposition of PDA complex.


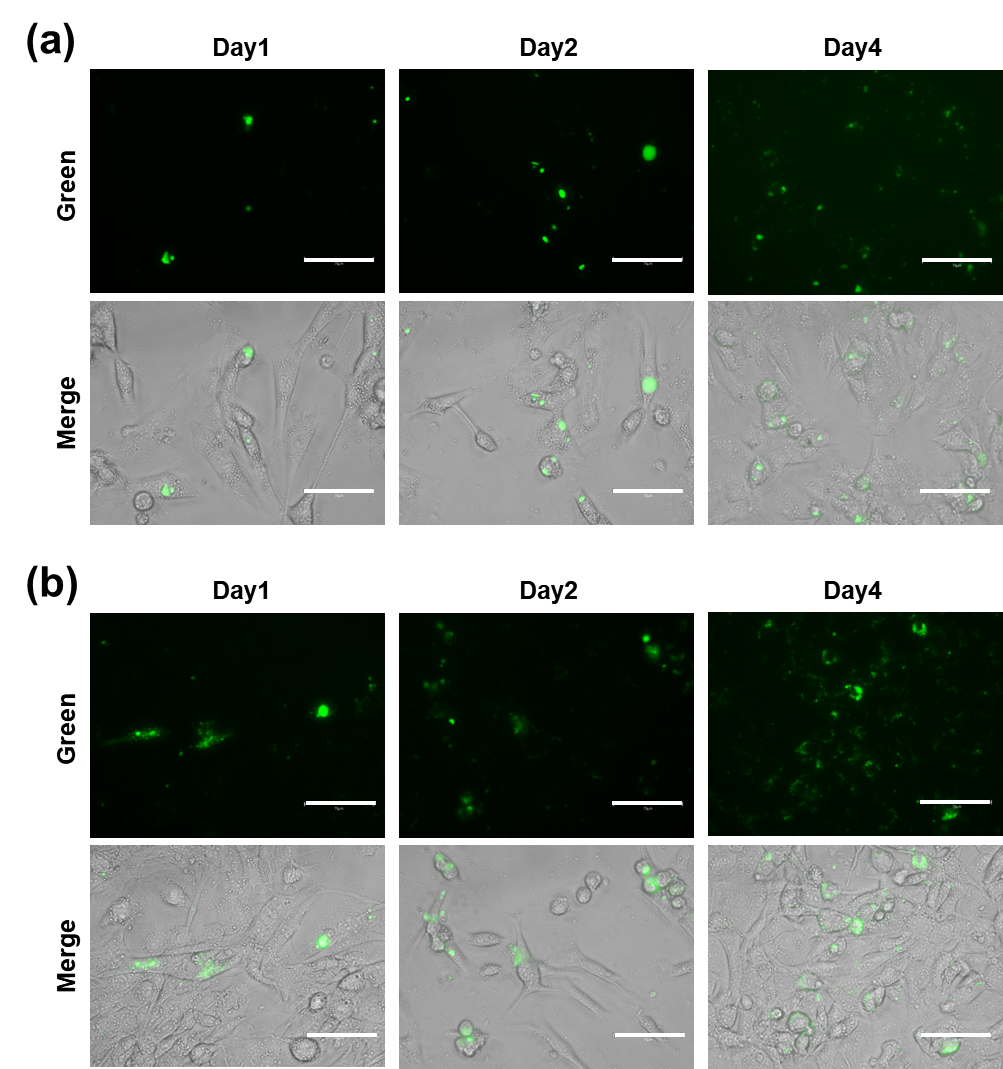


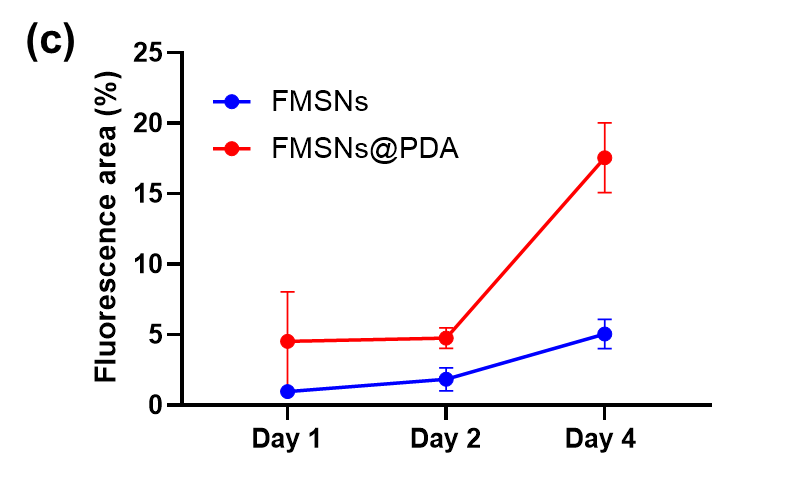


**Fig. S7.** Fluorescence images of 3T3-L1 after incubation with (a) FMSNs and (b) FMSNs@PDA. (c) Quantitative fluorescence analysis of cellular uptake for FMSNs and FMSNs@PDA.

**Table S1.** Fitted parameter values of K-P, Hill and BiDoseResp model for the release data

| **Drug vehicles** | **FMSNs-EGCG** | | | | | |
| --- | --- | --- | --- | --- | --- | --- |
|  | K-P model | | | Hill model | | |
| pH 4.0 | k_R_ = 0.088 | n = 0.62 | R^2^ = 0.971 | $\gamma$ = 2.47 | t_1/2_ = 2.92 | R^2^ = 0.996 |
| pH 5.5 | k_R_ = 0.094 | n = 0.50 | R^2^ = 0.981 | $\gamma$ = 1.76 | t_1/2_ = 3.84 | R^2^ = 0.995 |
| pH 7.4 | k_R_ = 0.082 | n = 0.47 | R^2^ = 0.955 | $\gamma$ = 2.07 | t_1/2_ = 2.07 | R^2^ = 0.991 |
|  | **FMSNs@PDA-EGC** | | | | | |
|  | K-P model | | | BiDoseResp model | | |
| pH 4.0 | k_R_ = 0.081 | n = 0.62 | R^2^ = 0.859 | h_1_ = 2.324 | h_2_ = 0.390 | R^2^ = 0.992 |
| pH 5.5 | k_R_ = 0.039 | n = 0.82 | R^2^ = 0.966 | h_1_ = 0.700 | h_2_ = 0.463 | R^2^ = 0.995 |
| pH 7.4 | k_R_ = 0.063 | n = 0.65 | R^2^ = 0.921 | h_1_ = 3.260 | h_2_ = 0.313 | R^2^ = 0.993 |

**Table S2.** Zeta potentials of FMSNs@PDA in PBS at different pH levels by ELS

| pH | Zeta potential (mV) |
| --- | --- |
| 3 | 19.29 ± 2.48 |
| 5 | -19.37 ± 2.84 |
| 7 | -33.98 ± 4.21 |
| 9 | -40.33 ± 3.47 |
| 11 | -63.31 ± 3.34 |
